# *Atractylodes lancea* (Thunb.) DC. [Asteraceae] Rhizome-Derived Exosome-like Nanoparticles Suppress Lipopolysaccharide-Induced Inflammation by Reducing Toll-like Receptor 4 Expression in BV-2 Murine Microglial Cells

**DOI:** 10.3390/ph18081099

**Published:** 2025-07-24

**Authors:** Mizusa Hyodo, Kei Kawada, Tomoaki Ishida, Yuki Izawa-Ishizawa, Ryoko Matoba, Rina Okamoto, Kohei Jobu, Io Horikawa, Fuka Aizawa, Kenta Yagi, Takahiro Niimura, Yayoi Kawano, Shinji Abe, Yukihiro Hamada, Mitsuhiro Goda, Keisuke Ishizawa

**Affiliations:** 1 Department of Clinical Pharmacology and Therapeutics, Tokushima University Graduate School of Biomedical Sciences, 3-18-15, Kuramoto-cho, Tokushima 770-8503, Japan; c202456013@tokushima-u.ac.jp (M.H.); ishizawa-yuki@shikoku-u.ac.jp (Y.I.-I.); horikawa.io@tokushima-u.ac.jp (I.H.); f_aizawa@tokushima-u.ac.jp (F.A.); yagi.kenta@med.shimane-u.ac.jp (K.Y.); niimura@tokushima-u.ac.jp (T.N.); mitsuhirogoda@hiroshima-u.ac.jp (M.G.); ishizawa@tokushima-u.ac.jp (K.I.); 2 Department of Pharmacy, Tokushima University Hospital, 2 Chome-50-1 Kuramoto-cho, Tokushima 770-8503, Japan; 3 Department of Clinical Pharmacy, Nagoya City University Graduate School of Pharmaceutical Sciences, 3-1, Tanabe-dori, Mizuho-ku, Nagoya 467-8603, Japan; y.kawano@phar.nagoya-cu.ac.jp; 4 Department of Health and Nutrition, Faculty of Human Life Science, Shikoku University, 123-1, Ebishiko-no, Furukawa, Ojin-cho, Tokushima 771-1192, Japan; 5 Department of Clinical Pharmacy Practice Pedagogy, Tokushima University Graduate School of Biomedical Sciences, 3-18-15, Kuramoto-cho, Tokushima 770-8503, Japan; c402104082@tokushima-u.ac.jp (R.M.); c402104001@tokushima-u.ac.jp (R.O.); ashinji@tokushima-u.ac.jp (S.A.); 6 Department of Pharmacy, Kochi Medical School Hospital, 185-1 Okochokohasu, Nankoku 783-0043, Japan; jm-kouheij@kochi-u.ac.jp (K.J.); hamada_yukihiro@kochi-u.ac.jp (Y.H.); 7 Clinical Research Center for Developmental Therapeutics, Tokushima University Hospital, 2-50-1 Kuramoto, Tokushima 770-8503, Japan; 8 Department of Pharmacy, Shimane University Hospital, 89-1 Enya-cho, Izumo 693-8501, Japan; 9 Department of Pharmacotherapy, Graduate School of Biomedical and Heath Sciences, Hiroshima University, Kasumi 1-2-3, Minami-ku, Hiroshima 734-8553, Japan

**Keywords:** *Atractylodes lancea* rhizome, microglia, exosome-like nanoparticle, neuroinflammation, RNA sequencing, ingenuity pathway analysis, Toll-like receptor 4, BV-2, nitric oxide

## Abstract

**Background/Objectives:** *Atractylodes lancea* (Thunb.) DC. [Asteraceae] (ALR)-derived exosome-like nanoparticles (ALR-ELNs) exhibit anti-neuroinflammatory effects in microglial cells. However, the associated mechanisms and pathways are unknown. We aimed to characterize the effects of ALR-ELNs on inflammatory responses of BV-2 microglial cells to lipopolysaccharide (LPS) using RNA sequencing. **Methods:** ALR-ELNs were fractionated from ALR. BV-2 microglial murine cells were stimulated with LPS after treatment with ALR-ELNs. RNA sequencing was performed to analyze variations in mRNA levels. Ingenuity pathway analysis (IPA) was performed to investigate the mechanism of action of ALR-ELNs. mRNA expression was assessed using real-time quantitative polymerase chain reaction (qPCR). **Results:** The expression of 651 genes was downregulated, whereas that of 1204 genes was upregulated in LPS-stimulated BV2 cells pretreated with ALR-ELNs. The IPA showed that the effects of ALR-ELNs on inflammation took place through pathogen-influenced signaling. Network analysis via IPA showed that the Toll-like receptor (TLR) is involved in the suppression of inflammation by ALR-ELNs. The qPCR analysis showed that pretreatment with ALR-ELNs significantly reduced *TLR4* mRNA expression. **Conclusions:** ALR-ELNs suppress the release of inflammatory mediators by downregulating *TLR4* expression, which is a novel mechanism by which ALR-ELNs act on microglia. Identifying active ingredients in ALR-ELNs that downregulate *TLR4* expression can advance the development of therapeutic drugs for neuroinflammatory diseases.

## 1. Introduction

Cell–cell communication is a physiological mechanism that contributes to the maintenance of tissue function and homeostasis. Plant-derived exosome-like nanoparticles (ELNs) can enable cross-species cellular communication by delivering molecular information to recipient cells upon ingestion [1]. These nanoparticles derived from edible plants can cross species boundaries to transfer molecular signals to the recipient cells upon ingestion [2]. ELNs derived from edible plants (such as grapes, grapefruit, ginger, and carrots) exert anti-inflammatory effects and can help maintain intestinal homeostasis [3]. *Citrus limon*-derived ELNs exhibit antioxidant and antitumor effects [4,5]. Ginger-derived ELNs can prevent the development of liver-related diseases, including those caused by alcohol consumption [6]. Collectively, these findings have opened up new perspectives on the therapeutic potential of natural/food compounds [7].

*Atractylodes lancea* (Thunb.) DC. [Asteraceae] is a perennial herbaceous plant native to China, Japan, and Korea; *A. lancea* rhizome (ALR) is widely used in traditional Chinese medicine [8]. It has long been valued for its medicinal properties. ALR is used to treat rheumatic diseases, digestive disorders, night blindness, and influenza [9]. It exerts anticancer, anti-obesity, and anti-inflammatory effects owing to its sesquiterpene, sesquiterpenoid, polyethylene alkyne, and phytosterol content [8,9]. A previous study reported the successful extraction of ELNs from ALR and showed that ALR-derived exosome-like nanoparticles (ALR-ELNs) were taken up by microglial cells, suppressing lipopolysaccharide (LPS)-stimulated inflammation [10].

Neuroinflammation causes neurological damage, and it has been implicated in the development of several neurodegenerative diseases such as Alzheimer’s disease (AD), Parkinson’s disease, Huntington’s disease, and multiple sclerosis [11]. Microglial cells are specialized immune cells that comprise approximately 10–20% of cells in the central nervous system and are involved in initiating the innate immune response [12]. In response to neuronal injury, infection, or stress, microglia become activated and secrete various pro-inflammatory molecules such as tumor necrosis factor (TNF)-α, interleukin (IL)-1β, IL-6, nitric oxide (NO), reactive oxygen species (ROS), and prostaglandin E2 (PGE2) [13]. Neuroinflammation, a major cause of neurodegenerative diseases, can result from aberrant microglial activation [14]. Therefore, understanding the anti-inflammatory effects of ALR-ELNs in microglia is important in the development of therapeutic agents for neuroinflammatory diseases. In the present study, we aimed to characterize the effects of ALR-ELNs on the inflammatory response of BV-2 microglial cells to LPS.

## 2. Results

### 2.1. ALR-ELNs Suppressed LPS-Induced NO Production

The average diameter of the ALR-ELN population was 297.4 ± 46.7 nm, and the average zeta potential was −36.4 ± 3.7 mV (Supplementary Figure S1a,b). These data were obtained using a Zetasizer Ultra (Malvern Panalytical Ltd., Malvern, UK) with ZS Xplorer software version 3.31 and represent one of three independently prepared batches.

**Figure 1:**
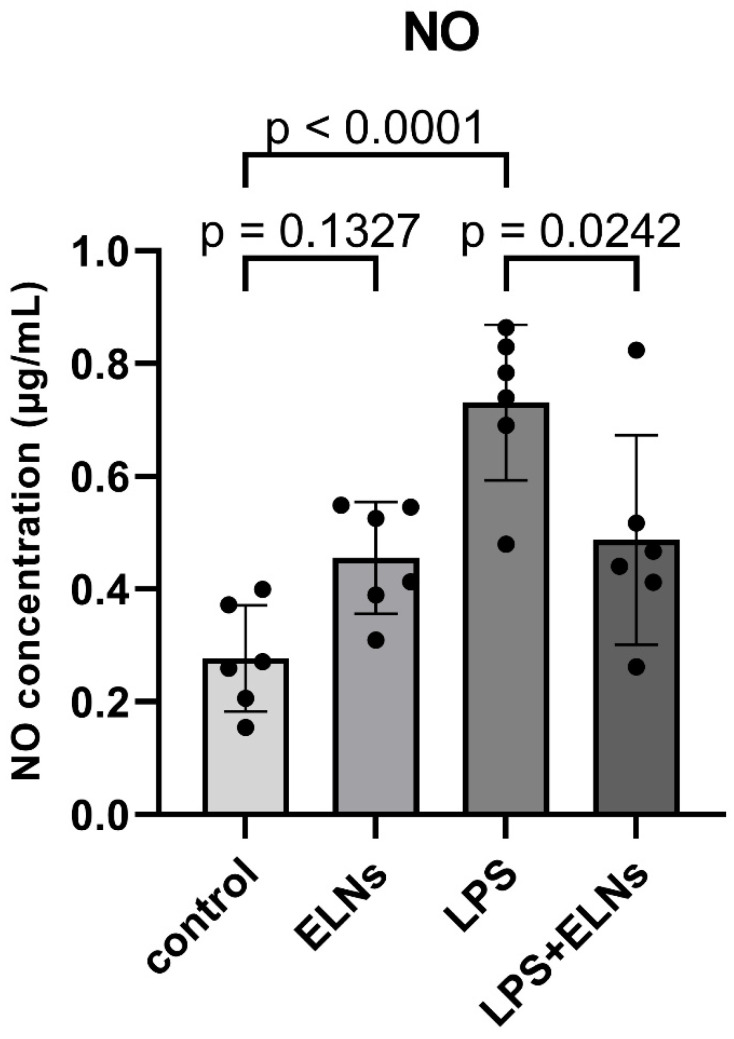
Effects of ALR-ELN treatment on LPS-induced nitric oxide (NO) production. Values are expressed as the mean ± standard deviation: LPS vs. control, LPS+ELN vs. LPS, using one-way ANOVA followed by Tukey’s test.

To examine the anti-inflammatory effects of ALR-ELNs, we measured the production of NO, a mediator of inflammatory responses. The NO level in the LPS group was significantly higher than that in the control group (*p* < 0.0001), and it was significantly lower in the LPS+ELN group than that in the LPS group (*p* = 0.0242) (Figure 1). This finding suggests that ALR-ELNs can suppress LPS-induced inflammatory responses.

### 2.2. ALR-ELNs Altered the Expression Levels of Multiple Genes

**Figure 2:**
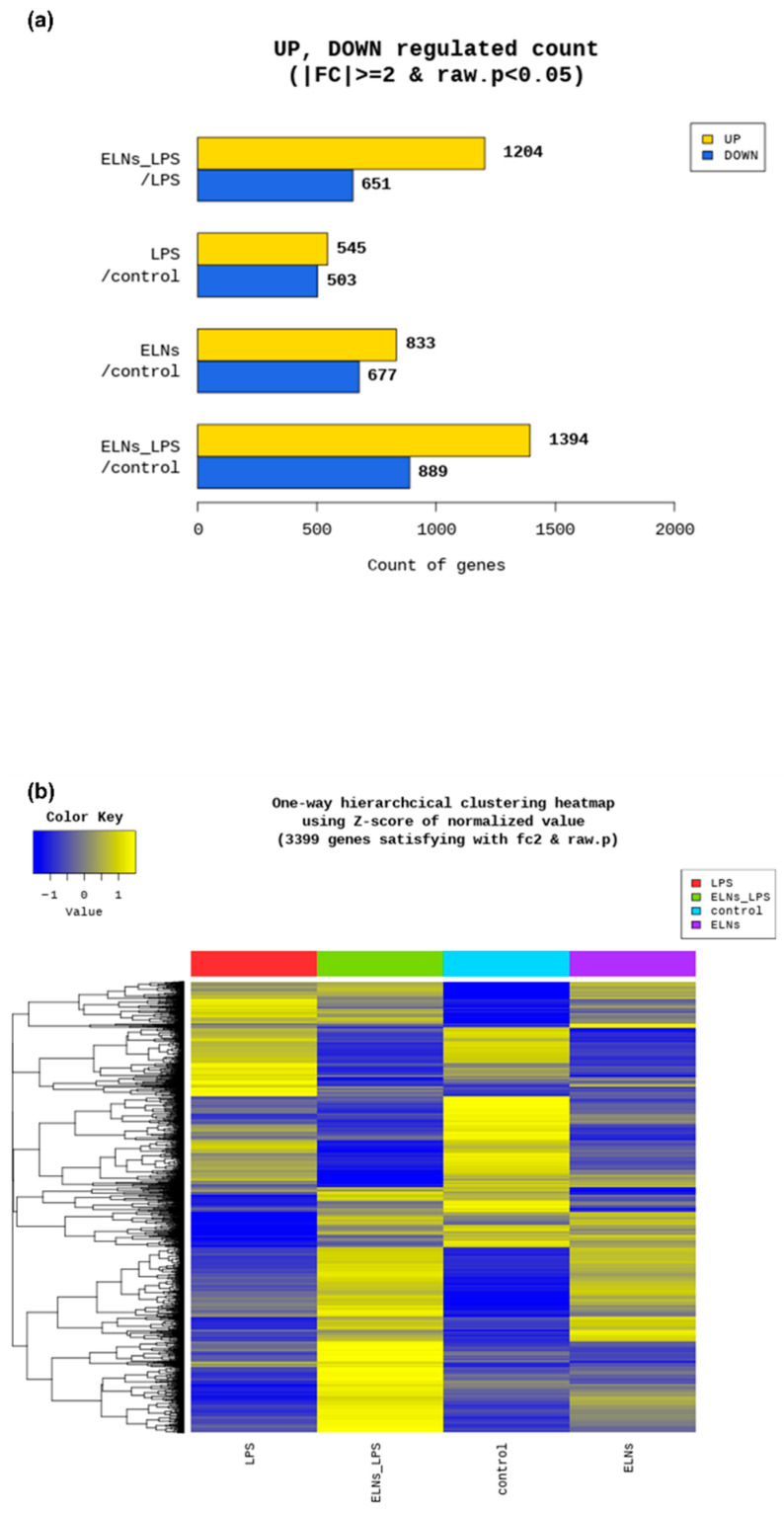
Global gene expression changes following ALR-ELN treatment. (**a**) Upregulated and downregulated gene counts by fold change and *p*-value. The number of upregulated and downregulated genes is shown based on the fold change and *p*-value in a comparison pair. (**b**) Heatmap showing the results of hierarchical clustering analysis (distance metric = Euclidean distance, linkage method = complete). The figure graphically represents the similarity of gene expression patterns between samples.

We performed RNA sequencing to examine the effect of ALR-ELNs on gene expression levels in BV-2 cells. The expression of 1204 genes was upregulated, and that of 651 genes was downregulated in the LPS+ELN group compared with those in the LPS group (|FC| ≥ 2 and raw. *p* < 0.05: LPS+ELNs vs. LPS) (Figure 2a). The heatmap shows that genes upregulated in the LPS group were downregulated in the LPS+ELN group, whereas genes downregulated in the LPS group were upregulated in the LPS+ELN group. This finding indicates an overall inverse relationship in the gene expression levels between the groups. A similar inverse relationship regarding gene expression changes was observed between the ELN and control groups (Figure 2b).

### 2.3. Canonical Pathway and Gene Ontology Enrichment Analyses

**Figure 3:**
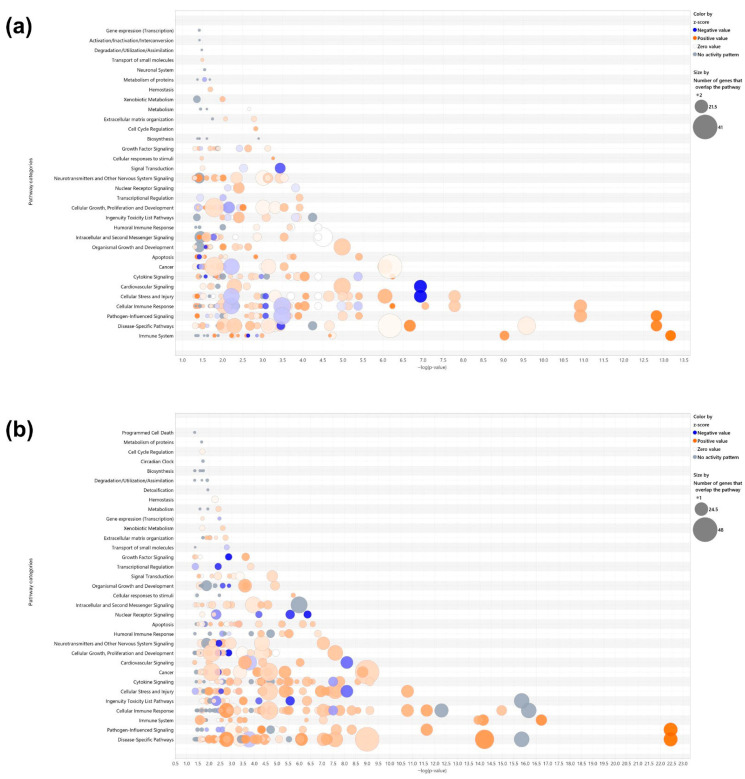

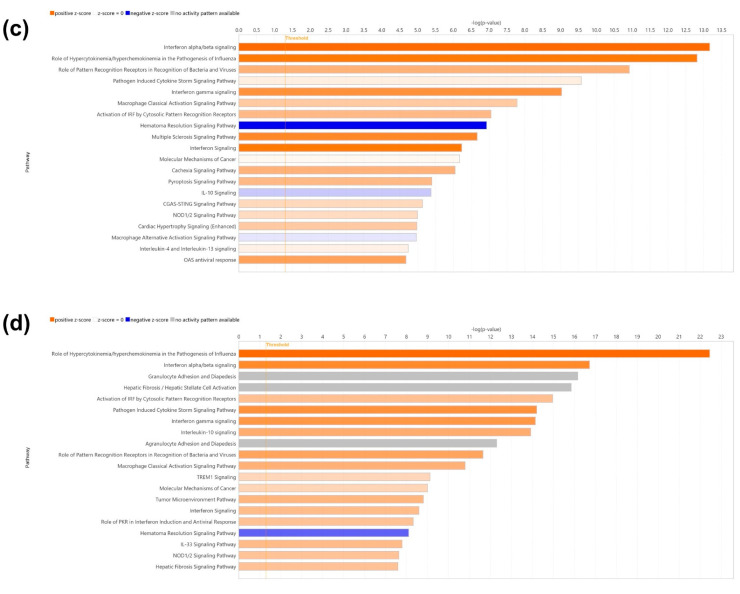
Effects of ALR-ELN treatment on immune response and signaling pathway activation. Bubble charts that show significantly enriched canonical pathway categories (Y axis) are listed according to the right-tailed Fisher’s exact test *p*-values (X axis; −log (*p*-value) > 1.3) as shown: (**a**) LPS+ELN vs. LPS and (**b**) ELN vs. control. Orange circles: positive-value z-score; blue circles: negative-value z-score; gray circles: zero-value z-score. Detailed information on molecules belonging to each pathway category is provided in Supplementary Table S1 (LPS+ELNs vs. LPS) and Table S2 (ELNs vs. control). The vertical axis represents the pathway classification category, and the size of each circle indicates the number of genes that overlap in each pathway. Horizonal bars indicate the top 20 significantly enriched canonical pathways (Y axis) listed according to their *p*-values (X axis; −log (*p*-value) > 1.3), (**c**) LPS+ELN vs. LPS and (**d**) ELN vs. control. Orange bars, predicted pathway activation z-score; blue bars, predicted pathway inhibition; gray bars, no activity prediction; white bars, pathways with z-scores at or very close to 0 or if there are fewer than four analysis-ready molecules, resulting in a z-score that cannot be calculated (z-score = NaN), respectively. Z-scores that are greater than or equal to 2 represent predictions of activation, whereas those less than or equal to −2 represent predictions of inhibition.

Canonical pathway analysis was conducted using ingenuity pathway analysis (IPA) based on differentially expressed genes (DEGs) with |fold change| ≥ 3 and *p* < 0.05. In the LPS+ELN vs. LPS comparison, significantly enriched pathways included interferon signaling, Toll-like receptor signaling, and IL-6 signaling (*p* < 0.01). In the ELNs vs. control comparison, enrichment was observed in interferon alpha/beta signaling and the activation of IRF by cytosolic pattern recognition receptors. The top 20 pathways for each comparison are shown in Figure 3a–d. Detailed pathway information, including z-scores, *p*-values, and gene counts, is presented in Supplementary Tables S1 and S2. In addition to inflammation-related pathways, enrichment was also observed in other biological pathways. In the LPS+ELN vs. LPS comparison, these included Molecular Mechanisms of Cancer, Cachexia Signaling Pathway, and Cardiac Hypertrophy Signaling. In the ELN vs. control comparison, Tumor Microenvironment Pathway, Hepatic Fibrosis Signaling, and Hematoma Resolution Signaling Pathway were enriched.

Gene Ontology (GO) enrichment analysis based on DEGs from the LPS+ELNs vs. LPS comparison showed a significant overrepresentation of the following terms: “regulation of response to cytokine stimulus” (gene count = 42, *p* < 0.001; Supplementary Table S1), “excitatory synapse” (gene count = 18, *p* < 0.001; Supplementary Table S2), and “nucleotidyltransferase activity” (gene count = 12, *p* < 0.01). To visualize the global effects of ALR-ELNs in unstimulated cells, IPA was newly performed for the ELNs vs. control comparison, and the corresponding bubble plots and bar graphs have been added as Figure 3b,d, with detailed data provided in Supplementary Table S2.

### 2.4. TLR Is Involved in the Action of ALR-ELNs in LPS-Induced Inflammation

**Figure 4:**
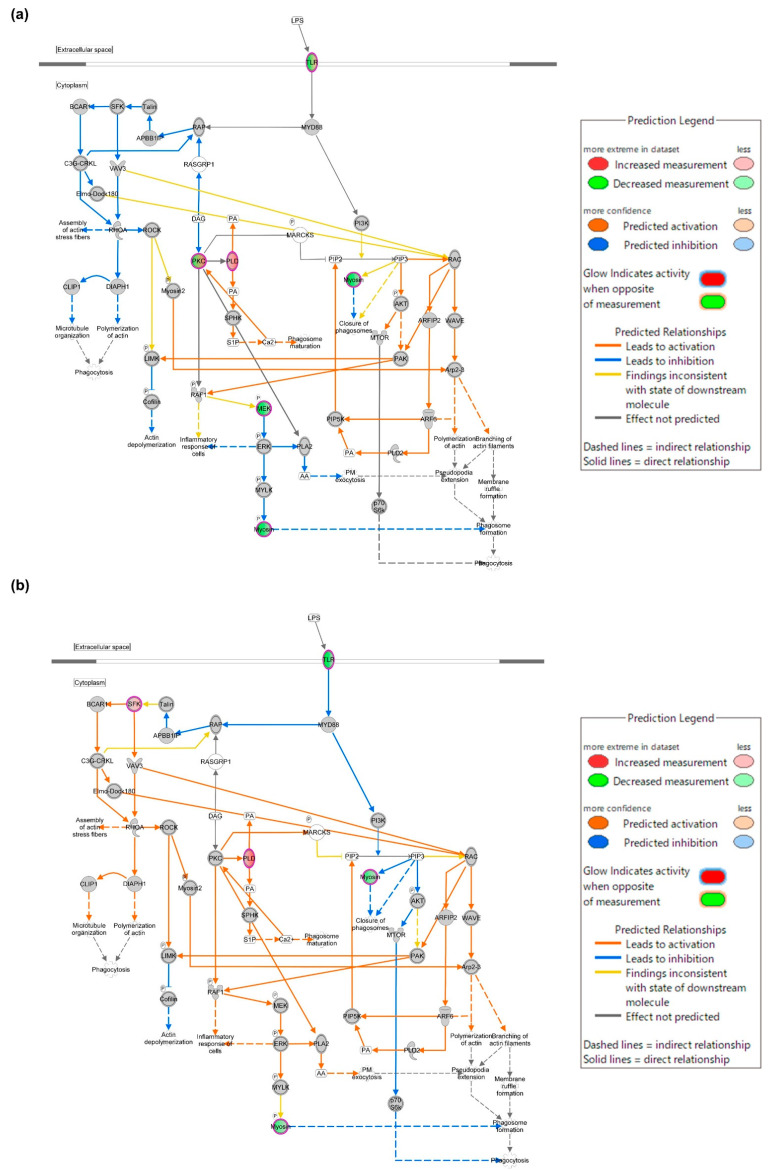
Mechanism through which ALR-ELNs suppress LPS-induced inflammation. Phagosome formation network regulated by TLR. Experimental upregulated (pink) and downregulated (green) DEGs in LPS-stimulated BV-2 cells pretreated with ALR-ELNs. Predicted upregulated and downregulated genes according to the molecular activity predictor are depicted in orange and blue, respectively. (**a**) LPS+ELN vs. LPS and (**b**) ELN vs. control. A double circle indicates that the molecule belongs to a family.

To investigate the genes through which ALR-ELNs affect LPS-induced inflammation, we performed regulator effect analysis via IPA (|FC| ≥ 3 and raw. *p* < 0.05: LPS+ELNs vs. LPS, ELNs vs. control). The mRNA expression of *TLR* decreased in the LPS+ELN group (Figure 4a) compared with that in the LPS group. The mRNA expression of *TLR* also decreased in the ELN groups compared with that in the control group (Figure 4b).

### 2.5. ALR-ELNs Reduced the mRNA Expression of TLR4

**Figure 5:**
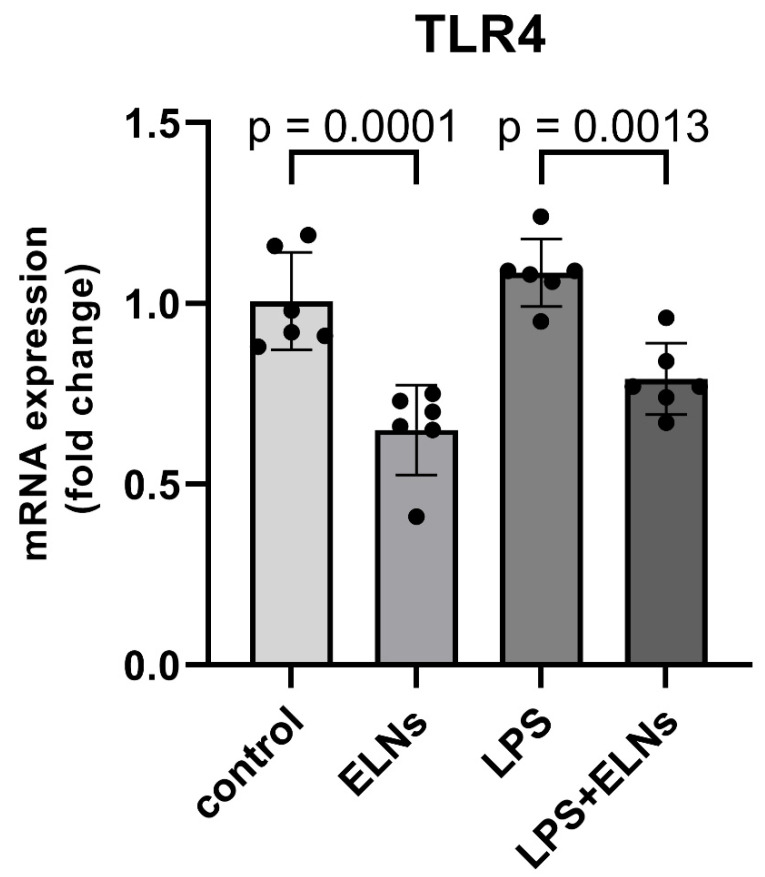
Effects of ALR-ELN treatment on LPS-induced *TLR4* mRNA level. *TLR4* mRNA expression levels. Values are expressed as mean ± standard deviation: LPS+ELN vs. LPS, ELN vs. control using one-way ANOVA followed by Tukey’s test.

The effect of ALR-ELNs on *TLR4* mRNA expression was examined using a real-time quantitative polymerase chain reaction (qPCR). The LPS+ELN group showed significantly lower *TLR4* mRNA expression than the LPS group (LPS+ELNs vs. LPS: *p* = 0.0013). Similarly, the ELN group showed significantly lower *TLR4* mRNA expression than the control group (ELNs vs. control: *p* = 0.0001) (Figure 5).

## 3. Discussion

In this study, omics analysis combining RNA sequencing and IPA was performed to elucidate the mechanism of the anti-inflammatory action of ALR-ELNs on BV-2 microglial cells. RNA sequencing revealed changes in the expression of several genes in BV-2 cells upon ALR-ELN treatment. IPA showed that pathogen-influenced signaling was the main pathway of action of ALR-ELNs. Furthermore, network analysis indicated that ALR-ELN treatment decreased the mRNA expression of *TLR*. Finally, qPCR analysis revealed that ALR-ELN treatment decreased *TLR4* mRNA expression. The transcriptome analysis showed that ALR-ELNs affect the expression of TLR4, which is important in the inflammatory response to LPS stimulation.

A previous study reported the successful extraction of exosome-like nanoparticles from ALR and showed that ALR-ELNs were taken up by microglial cells and that they suppressed LPS-stimulated inflammation [10]. Furthermore, ALR-ELN treatment significantly decreased the mRNA levels of the inflammation-related genes *IL-1β*, *IL-6*, inducible nitric oxide synthase (*iNOS*), and C-X-C motif chemokine 10 (*CXCL10*) in BV-2 cells, which were increased following LPS exposure. In contrast, it significantly increased the levels of the anti-inflammatory genes heme oxygenase 1 (*HMOX1*), interferon regulatory factor 7 (*IRF7*), chemokine (C-C motif) ligand 12 (*CCL12*), and immune-responsive gene 1 (*IRG1*). These results indicate that ALR-ELNs exert their anti-neuroinflammatory effect by acting on multiple inflammation-related mediators; however, the detailed mechanism of action remains to be fully elucidated. Plant-derived ELNs are generally internalized by recipient mammalian cells via endocytic pathways, such as phagocytosis or micropinocytosis [3,15]. In our previous study [10], we confirmed that ALR-ELNs are taken up by BV-2 microglial cells, as demonstrated by the intracellular fluorescence of labeled nanoparticles observed using confocal microscopy. Although the specific uptake mechanism in the present study was not directly investigated, it is likely that ALR-ELNs are internalized through receptor-mediated or endocytotic routes. The influence of ALR-ELNs on the function of multiple inflammation-related mediators suggests that the mechanism of their anti-inflammatory action in microglial cells is complex. In addition to their anti-inflammatory properties, ALR-ELNs were found to modulate several signaling pathways not directly related to inflammation. As shown in Figure 3b,d, the treatment of naïve BV-2 microglial cells with ALR-ELNs alone led to the significant enrichment of canonical pathways such as EIF2 signaling, mTOR signaling, oxidative phosphorylation, and cell-cycle regulation. These pathways are typically involved in protein synthesis, cellular metabolism, mitochondrial function, and proliferation. Although no strong activation or inhibition (as indicated by z-scores) was detected for most of these pathways, their enrichment suggests that ALR-ELNs may exert broader biological effects beyond immune modulation. Notably, “Molecular Mechanisms of Cancer” and “Tumor Microenvironment Pathway” were also among the pathways enriched in the ELNs vs. control comparison (Supplementary Table S2). While the z-scores of these pathways were close to neutral, their presence warrants attention. The dysregulation of pathways such as EIF2 signaling and mTOR signaling has been implicated in various pathological conditions, including tumorigenesis, neurodegenerative diseases, and metabolic disorders [16,17]. These findings highlight the importance of evaluating not only the therapeutic efficacy but also the potential off-target effects of ALR-ELNs in future safety and toxicology studies. Long-term or systemic administration, in particular, should be approached with caution until further data are available regarding the specificity and biological safety of ALR-ELNs.

RNA sequencing analysis showed that ALR-ELN treatment downregulated the expression levels of 651 genes and upregulated the expression of 1204 genes in LPS-stimulated BV-2 cells. These results suggest that ALR-ELN treatment mostly downregulated gene expression in LPS-stimulated BV-2 cells. IPA can show the biological significance of complex omics data by predicting the activation or inhibition of relevant signaling and metabolic pathways from RNA sequencing data [18]. The IPA results showed that ALR-ELN treatment suppressed the pathogen-influenced signaling pathway and downregulated TLR expression. A study examining the effects of blueberry-derived ELNs on endothelial cells using IPA showed that these ELNs suppress TLR signaling [19]. These findings suggest that TLRs play an important role in the anti-inflammatory effects of plant-derived ELNs.

LPS induces inflammatory responses by binding to and activating TLR4 expressed on cell membranes [20]. LPS-stimulated TLR4 activates immune cell signaling pathways and induces the expression of inflammatory genes and cytokines [21]. TLR4 activation plays an important role in the progression of diseases such as neuroinflammation, AD, and dementia [22,23,24]. In particular, the activation of the TLR4 signaling pathway, which promotes the release of inflammatory cytokines, such as TNF-α, IL-1, IL-6, and iNOS [25], triggers various neuroinflammatory responses. The development of AD is thought to be driven by the generation and deposition of amyloid-β (Aβ) [23]. Notably, TLR4 activation can reduce the CD36-mediated phagocytosis of Aβ [24]. Moreover, TLR4-mediated inflammatory responses may lead to synaptic dysfunction and neuronal loss, which are key features of dementia [25]. Conversely, the inhibition of *TLR4* expression may slow the progression of neuroinflammation, AD, and related dementias. In addition, inhibiting the expression of TLR4, a receptor on microglia, reduces microglial activation and the release of inflammatory cytokines, including IL-6, TNF-α, and IL-1β, alleviating the chronic neuroinflammation associated with AD and other dementias [26]. Inhibiting TLR4 expression has also been found to block the Aβ oligomer-mediated microglial cell activation pathway and reduce the inflammatory response to Aβ accumulation [24]. Finally, TLR4 inhibition may attenuate tau-related pathology in AD by regulating the neuronal autophagy machinery [26]. These findings suggest that the inhibition of TLR4 expression may be effective against neuroinflammation, AD, and dementia.

In the present study, we observed that ALR-ELN treatment mitigated the inflammation-induced increase in TLR4 expression and its downstream signaling components. Notably, *TLR4* expression was significantly downregulated not only in the LPS+ELN group but also in cells treated with ALR-ELNs alone, even in the absence of LPS stimulation (Figure 5). These findings suggest that ALR-ELNs may exert a basal suppressive effect on TLR4 gene transcription, independent of inflammatory challenge. One plausible mechanism for this effect involves the delivery of bioactive molecules—such as plant-derived microRNAs or 7-methoxycoumarin—encapsulated within ALR-ELNs [10]. Previous studies have demonstrated that edible plant-derived exosome-like nanoparticles can modulate host immune responses, including through microRNA-mediated gene regulation [27,28]. In a previous study [10], we identified several microRNAs within ALR-ELNs that have been predicted to target key inflammation-related genes, including *TLR4*. These microRNAs may suppress *TLR4* expression either directly or by modulating upstream transcriptional regulators, contributing to the anti-inflammatory profile observed. Importantly, our current findings suggest that ALR-ELNs not only suppress the inflammatory response downstream of *TLR4*, as previously reported [10], but also actively downregulate *TLR4* expression itself, positioning them as potential upstream modulators of immune activation. This distinction indicates that the anti-inflammatory effects of ALR-ELNs may be partially mediated through the transcriptional repression of pattern recognition receptors, offering a mechanistic advantage in the modulation of neuroinflammation. Notably, ALR-ELNs were administered as a preventive treatment prior to LPS exposure in this study. Whether similar suppressive effects on *TLR4* expression and inflammatory signaling can be achieved when ALR-ELNs are administered after the onset of inflammation remains to be determined. Future studies should explore the therapeutic window and durability of the effects of ALR-ELN in models of established neuroinflammation. Furthermore, the identification and functional validation of the specific molecular components within ALR-ELNs responsible for *TLR4* downregulation will be critical in translating these findings into potential therapeutic strategies. Such insights could facilitate the development of novel interventions targeting TLR4-mediated neuroinflammatory pathways in diseases such as Alzheimer’s disease, multiple sclerosis, and Parkinson’s disease.

This study has certain limitations. First, the possibility that pathways other than those involving TLR4 play a role in the anti-inflammatory effects of ALR-ELNs cannot be ruled out. Second, we previously identified several microRNAs (ath-miR166f, ath-miR162a-5p, and ath-miR162b-5p) and 7-methoxycoumarin as candidate components for the active ingredients of ALR-ELNs [10]. The mechanism through which these potential active ingredients function remains unclear. Third, whether the effects of ALR-ELN treatment can be explained solely by the decrease in TLR4 expression is unclear as an increase in the expression of anti-inflammatory genes has also been observed in a previous study [10]. Further studies are required to elucidate the mechanism behind the anti-neuroinflammatory effects of ALR-ELNs.

This is the first study to demonstrate that TLR4 is involved in the anti-inflammatory effect of ALR-ELNs on BV-2 microglia cells. We believe that this study will contribute to further research on the pharmacokinetics of plant-derived ELNs. Once the mechanism by which ALR-ELNs exert their anti-inflammatory effects is uncovered, ALR-ELNs may represent a promising candidate for anti-neuroinflammatory interventions.

## 4. Materials and Methods

### 4.1. Isolation and Characterization of A. lancea Exosome-like Nanoparticles

Tsumura (Tokyo, Japan) supplied the ALR samples, which were authenticated according to the Japanese Pharmacopoeia’s 18th edition. Using a previously described method [10,29], exosome-like nanoparticles were extracted from ALR extracts through a process combining differential centrifugation and membrane filtration. The procedure involved preparing ALR extracts by boiling 20 g of dried rhizome in 400 mL of water for 30 min. The resulting extract was subjected to filtration and sequential centrifugation at 8000× *g* for 5 min and then at 15,000× *g* for 20 min using an Eppendorf 5417R centrifuge (Eppendorf SE, Hamburg, Germany). The supernatant was then filtered through a 0.8 µm Minisart NML syringe filters (#16592; Sartorius AG, Göttingen, Germany), and an exoEasy Maxi Kit (QIAGEN, Hilden, Germany) was used to further purify the exosome-like nanoparticles. The isolated ALR-ELNs were preserved at −70 °C for future experiments. From 20 g of dried ALR, the total yield of ALR-ELNs was approximately 10.4 ± 3.0 mg. The size and zeta potential of ALR-ELNs were analyzed using a Zetasizer Ultra (Malvern Panalytical Ltd., Enigma Business Park, Grovewood Road, Malvern, UK). The average particle size was 297.4 ± 46.7 nm, and the zeta potential was −36.4 ± 3.7 mV (Supplementary Figure S1a,b). These values represent the results for one of three independently prepared batches.

### 4.2. BV-2 Microglial Cell Culture

AcceGen Biotechnology (Fairfield, NJ, USA) supplied BV-2 microglial cells (ABC-TC212S), which were grown in Dulbecco’s Modified Eagle Medium (DMEM; Nacalai Tesque, Inc., Kyoto, Japan) with 10% heat-inactivated fetal bovine serum (FBS; Serana Europe GmbH, Brandenburg, Germany) and 1% penicillin–streptomycin solution (FUJIFILM Wako Pure Chemical Co., Osaka, Japan). The cells were maintained at 37 °C in a 5% CO_2_ environment. To reduce the effect of FBS-derived exosomes, the supernatant was ultracentrifuged overnight at 110,000× *g* and 4 °C using micro ultracentrifuge (CS150FNX; Eppendorf Himac Technologies, Ibaraki, Japan), following an established protocol [10]. For qPCR analysis, BV-2 cells were seeded in six-well plates at 5 × 10^4^ cells per well. For the NO assay, the cells were plated in 48-well plates at 3 × 10^4^ cells per well. After a 24 h incubation period, the cells were exposed to ALR-ELNs (20 µg/mL) for 3 h and then challenged with 0.5 µg/mL LPS for 2 h.

### 4.3. Griess Assay

The Griess assay was used to determine NO levels by measuring nitrite (NO_2_^−^), which serves as an indicator of NO production after oxidation in aqueous solutions. A modified version of a previously established protocol [10] was used to conduct the assay. The process involved preparing Griess reagent A by combining sulfanilamide (FUJIFILM Wako Pure Chemical Co.) (1% *w*/*v*) and phosphoric acid (FUJIFILM Wako Pure Chemical Co.) (5% *v*/*v*); Griess reagent B was prepared by dissolving N-(1-naphthyl) ethylenediamine dihydrochloride (FUJIFILM Wako Pure Chemical Co.) (1% *w*/*v*) in ultrapure water. Equal parts of reagents A and B were mixed just before use. To quantify the nitrite, equal volumes (100 µL each) of cell culture supernatant and the prepared Griess reagent were combined in a 96-well microplate, and the mixture was incubated at room temperature (25–27 °C) for 20 min. The SpectraMax i3 microplate reader (Molecular Devices, San Jose, CA, USA) was used to measure absorbance at 540 nm. NO concentration was determined using a standard calibration curve, which was generated using serial dilutions of a nitrite ion standard solution (NO_2_^−^, 1000 mg/L; FUJIFILM Wako Pure Chemical Co.) in DMEM.

### 4.4. RNA Isolation, qPCR, and Gene Expression Profiling

The RNeasy Plus Mini Kit (QIAGEN) was employed to extract total RNA, adhering to the manufacturer’s instructions. A NanoDrop™ spectrophotometer (Thermo Fisher Scientific, Waltham, MA, USA) was used to evaluate RNA concentration and purity, with an A260/A280 ratio of approximately 2.0 indicating high-quality RNA. The PrimeScript™ RT reagent Kit with gDNA Eraser (Takara Bio, Shiga, Japan) was utilized for reverse transcription, following the manufacturer’s instructions. Genomic DNA was eliminated through gDNA Eraser treatment, and cDNA synthesis was performed using 1000 ng of total RNA. Reverse transcription was performed by slightly modifying a previously published method [10]; specifically, the incubation temperature was adjusted to enhance the cDNA yield. qPCR was performed using the SYBR™ Green qPCR Master Mix (Thermo Fisher Scientific, Waltham, MA, USA) with a StepOnePlus Real-Time PCR System (Applied Biosystems, Thermo Fisher Scientific). The reaction mixture (10 µL) comprised 0.5 µL each of 20 µM forward and reverse primers, 2 µL of single-stranded cDNA (50 ng), 5 µL of SYBR™ Green qPCR Master Mix, and 2 µL of RNase-free dH_2_O (Takara Bio). The thermal cycling conditions were as follows: 50 °C for 2 min and 95 °C for 10 min, followed by 40 cycles at 95 °C for 15 s and 60 °C for 1 min. To verify the primer specificity, melting curve analysis was performed at the end of the amplification process. The ΔΔCt method [30] was employed to calculate the relative gene expression, with *GAPDH* serving as the internal control. Before using the ΔΔCt method, PCR efficiency was assessed using a standard curve and was confirmed to be within the acceptable range of 90–110% (R^2^ > 0.99). All qPCR experiments were conducted in triplicate.

### 4.5. RNA Sequencing

Next-generation sequencing (NGS) techniques for transcriptome analysis offer highly detailed and sensitive gene expression profiles [31,32]. RNA sequencing was performed by Macrogen Japan Corp. (Tokyo, Japan).

### 4.6. RNA Extraction and Library Preparation

Total RNA was isolated using the RNeasy Plus Mini Kit (QIAGEN) according to the manufacturer’s protocol. RNA quality was assessed using an Agilent 4200 TapeStation system. The RNA integrity number (RIN) was 9.8 or higher for all samples (*n* = 4), confirming that high-quality RNA was obtained. The specific RIN values were as follows: control, 9.9; LPS, 10.0; ELNs, 9.9; and LPS+ELNs, 9.8. The rRNA ratio was 3.2 or higher for all samples, indicating that the integrity of ribosomal RNA was maintained. RNA quantification was performed using the Quant-iT RiboGreen RNA Assay Kit (Invitrogen, Waltham, MA, USA). One microgram of total RNA from each sample was used to prepare libraries with the TruSeq Stranded mRNA Library Prep Kit (Illumina, San Diego, CA, USA).

### 4.7. Sequencing and Quality Control

The libraries were sequenced using the Illumina platform with paired-end 101 bp sequences, and the raw data quality was assessed using FastQC v0.11.7 (http://www.bioinformatics.babraham.ac.uk/projects/fastqc/; accessed on 21 October 2024). Adapter removal and the trimming of low-quality bases were performed using Trimmomatic 0.38 (http://www.usadellab.org/cms/?page=trimmomatic; accessed on 21 October 2024).

### 4.8. Data Analysis

The trimmed reads were mapped to the mouse genome (mm10) using HISAT2 version 2.1.0 (https://ccb.jhu.edu/software/hisat2/index.shtml; accessed on 21 October 2024). Transcript assembly and expression quantification were performed using StringTie version 2.1.3b (https://ccb.jhu.edu/software/stringtie/; accessed on 21 October 2024) to identify novel and alternatively spliced transcripts in addition to known genes and transcripts. Fragments per kilobase of transcript per million mapped reads (FPKM) and transcripts per kilobase million (TPM) were used for expression normalization. DEGs were analyzed using edgeR (version 3.34.0) (a package of the statistical analysis software R (version 4.1.0)) with the following criteria: |fold change (FC)| ≥ 2 and exactTest raw *p*-value < 0.05. In total, four group comparisons were performed: LPS+ELN vs. LPS (1855 genes), LPS vs. control (1048 genes), ELN vs. control (1510 genes), and LPS+ELN vs. control (2283 genes). GO term enrichment analysis was performed using gProfiler (https://biit.cs.ut.ee/gprofiler/; accessed on 21 October 2024).

### 4.9. IPA

Canonical pathway analysis was performed using Ingenuity Pathway Analysis (IPA^®^; QIAGEN, Redwood City, CA, USA; https://digitalinsights.qiagen.com/products-overview/discovery-insights-portfolio/analysis-and-visualization/qiagen-ipa/; accessed on 22 January 2025) on DEGs from two comparisons: LPS+ELN vs. LPS and ELN vs. control [26]. DEGs were defined as those with |fold change| ≥ 3 and raw *p*-value < 0.05. Significantly enriched canonical pathways were visualized using bubble plots and bar graphs based on the −log(*p*-value) and z-score. IPA enables the identification of pathways in which a particular gene set is significantly enriched through enrichment analysis. Using the obtained DEG list, canonical pathway analysis and regulator effect analysis were performed. To extract the major pathways and network interactions, we analyzed genes that fulfilled the conditions of |FC| ≥ 3 and exactTest raw *p*-value < 0.05. The regulatory effect analysis was performed for two comparisons: LPS+ELN vs. LPS (Table S1) and ELN vs. control (Table S2). Tables S1 and S2 provide complete lists of significantly enriched canonical pathways for the comparisons of LPS+ELN vs. LPS and ELN vs. control, respectively, including *p*-values, z-scores, and gene counts for each pathway. GO enrichment analysis was conducted using Metascape on DEGs from the LPS+ELN vs. LPS comparison. Significantly overrepresented GO terms were defined as those with *p* < 0.01 and gene count ≥ 10.

Additionally, network analyses based on differentially expressed genes were performed via IPA to identify potential molecular interaction networks modulated by ALR-ELNs. The networks were generated using the “Pathway Explorer” and “Molecular Activity Predictor” functions. Two comparisons were assessed: LPS+ELN vs. LPS, and ELN vs. control. Only networks with a high relevance score and consistent z-score predictions were visualized (Figure 4a,b).

### 4.10. Statistical Analysis

All statistical analyses other than the analysis of RNA sequencing data were performed using EZR version 1.29 (Saitama Medical Center, Jichi Medical University, Saitama, Japan) [33]. Data are expressed as the mean ± standard deviation. One-way analysis of variance (ANOVA) was performed to examine the significance of the differences between treatments. Subsequently, multiple-group comparisons were performed using Tukey’s test. Statistical significance was set at *p* < 0.05.

## Data Availability

The original contributions presented in this study are included in the article/Supplementary Materials. Further inquiries can be directed to the corresponding author.

## Supplementary Materials

The following supporting information can be downloaded at https://www.mdpi.com/article/10.3390/ph18081099/s1, Figure S1: (a) Representative particle size distribution of ALR-derived exosome-like nanoparticles (ALR-ELNs), measured using Zetasizer Ultra. The average diameter was 297.4 ± 46.72 nm; (b) Zeta potential distribution of ALR-ELNs, measured by electrophoretic light scattering. The average zeta potential was −36.4 ± 3.70 mV. Tables S1 and S2: Detailed pathway information, including z-scores, *p*-values, and gene counts. Table S3: (a) Regulatory effect analysis of differentially expressed genes between the LPS+ELN and LPS treatment groups; (b) Regulatory effect analysis of differentially expressed genes between the ELN and control groups. Figure S2: (a) Top 10 biological process terms in the GO functional analysis; (b) Top 10 cellular component terms in the GO functional analysis; (c) Top 10 molecular function terms in the GO functional analysis.

## Abbreviations

The following abbreviations are used in this manuscript:

ALR	*Atractylodes lancea* rhizome
ANOVA	Analysis of variance
DEG	Differentially expressed gene
ELN	Exosome-like nanoparticle
IPA	Ingenuity pathway analysis
LPS	Lipopolysaccharide
NO	Nitric oxide
qPCR	Real-time quantitative polymerase chain reaction
TNF	Tumor necrosis factor
