# Supplementary material for: Atractylodes lancea (Thunb.) DC. [Asteraceae] Rhizome-Derived Exosome-like Nanoparticles Suppress Lipopolysaccharide-Induced Inflammation by Reducing Toll-like Receptor 4 Expression in BV-2 Murine Microglial Cells
Source: Pharmaceuticals (Basel). 2025 Jul 24;18(8):1099. doi: 10.3390/ph18081099 (PMC12389435; doi:10.3390/ph18081099)
Supplement: Supplementary file 1 [file pharmaceuticals-18-01099-s001.zip › Table S1.pdf]

Table S1. Molecules belonging to each pathway category (LPS+ELN vs. LPS).

**Ingenuity Canonical Pathways**	**-log(p-value)**	**Ratio**	**z-score**	**Molecules**
Interferon Alpha/Beta Signaling	13.2	0.239	3.153	ADAR, GBP2, IFI35, IFIT1, IFIT2, IFIT3, IRF7, ISG20, OAS1, OAS2, OAS3, SOCS1, SOCS3, STAT1, STAT2, USP18, XAF1
Role of Hypercytokinemia/hyperchemokinemia in the Pathogenesis of Influenza	12.8	0.209	2.828	CXCL10, EIF2AK2, IFIT2, IFIT3, IL10, IL1B, IL36G, IRF7, ISG20, OAS1, Oas1b, OAS2, OAS3, RIGI, S1PR1, STAT1, STAT2, TLR3
Role of Pattern Recognition Receptors in Recognition of Bacteria and Viruses	10.9	0.135	1.667	CLCF1, CLEC6A, EIF2AK2, IL10, IL1B, IL36G, IRF7, OAS1, Oas1b, OAS2, OAS3, PRKCB, PRKCG, PTX3, RIGI, TGFB2, TLR3, TLR8, TNFSF10, TNFSF13, TNFSF15
Pathogen Induced Cytokine Storm Signaling Pathway	9.58	0.0809	0.365	Ccl7, Ccl8, CCR3, CCR5, CIITA, CLCF1, COL15A1, COL23A1, CXCL10, CXCL16, DHX58, IL10, IL1B, IL1R1, IL21R, IL36G, IRF7, MAPK11, MYC, NLRC5, RIGI, SOCS3, STAT1, TGFB2, TLR3, TLR8, TNFSF10, TNFSF13, TNFSF15, ZBP1
Interferon Gamma Signaling	9.03	0.16	2.324	CIITA, GBP2, GBP4, GBP6, GBP7, IRF7, OAS1, OAS2, OAS3, SOCS1, SOCS3, STAT1, TRIM14, TRIM46, TRIM5
Macrophage Classical Activation Signaling Pathway	7.78	0.101	1.147	CIITA, CLCF1, CXCL10, GBP2, GBP4, IKBKG, IL10, IL1B, IL36G, PARP14, PARP9, SOCS1, SOCS3, STAT1, STAT2, TGFB2, TNFSF10, TNFSF13, TNFSF15
Activation of IRF by Cytosolic Pattern Recognition Receptors	7.05	0.169	1.508	ADAR, DHX58, IFIT2, IKBKE, IKBKG, IL10, IRF7, RIGI, STAT1, STAT2, ZBP1
Hematoma Resolution Signaling Pathway	6.93	0.0814	-2.4	ARG1, CLCF1, CX3CR1, HMOX1, IL10, IL1B, IL36G, MAPK11, MMP2, NQO1, PLAT, PTGER1, S1PR1, SOCS1, SOCS3, STAT1, TGFB2, THBS1, TNFSF10, TNFSF13, TNFSF15
Multiple Sclerosis Signaling Pathway	6.67	0.0856	2.524	CLCF1, IL10, IL1B, IL36G, MAPK11, PARP10, PARP12, PARP14, PARP16, PARP9, PLP1, RNF213, STAT1, TGFB2, TLR3, TLR8, TNFSF10, TNFSF13, TNFSF15
Interferon Signaling	6.23	0.222	2.828	IFI35, IFIT1, IFIT3, OAS1, SOCS1, STAT1, STAT2, TAP1
Molecular Mechanisms of Cancer	6.18	0.0479	0.156	ADCY6, ADORA2B, Aph1c, ARHGEF4, CCR3, CCR5, CCRL2, CELSR3, CNR2, CX3CR1, EDN1, EGF, GNAL, GPR146, GPR183, HDAC9, IL15RA, IL1B, IL1R1, IL21R, ITGA2B, ITGA3, ITGB4, ITGB5, MAP2K6, MAPK11, MMP2, MYC, P2RY12, PRKCB, PRKCG, PTGER1, PTGER2, RHOV, S1PR1, SHC3, SSTR5, SUCNR1, TGFB2, VIPR1, VIPR2
Cachexia Signaling Pathway	6.05	0.0652	1.633	ADCY6, CASP4, CLCF1, EIF2AK2, GDF15, IKBKE, IKBKG, IL1B, IL1R1, IL36G, LCN2, MALAT1, MAPK11, PRKCB, PRKCG, S1PR1, SOCS3, STAT1, STAT2, TGFB2, TLR8, TNFSF10, TNFSF13, TNFSF15
Pyroptosis Signaling Pathway	5.4	0.117	1.508	CASP4, GBP2, GBP4, GBP7, IL1B, IL1R1, KCNQ1OT1, MAPK11, PANX1, TLR3, TLR8
IL-10 Signaling	5.38	0.0909	-0.535	CCN4, CCR5, CREB3L4, HMOX1, IKBKE, IKBKG, IL10, IL1B, IL1R1, IL36G, MAP2K6, MAPK11, SOCS3, STAT1

CGAS-STING Signaling Pathway	5.14	0.0929	0.832	ATP6V0D2, CLCF1, IDO2, IKBKE, IKBKG, IL1B, IL36G, STAT1, TGFB2, TNFSF10, TNFSF13, TNFSF15, ZBP1
NOD1/2 Signaling Pathway	4.99	0.0794	0.775	CLCF1, IKBKE, IKBKG, IL1B, IL36G, IRF7, MAP2K6, MAPK11, RIGI, TGFB2, TLR3, TLR8, TNFSF10, TNFSF13, TNFSF15
Cardiac Hypertrophy Signaling (Enhanced)	4.98	0.0517	1.147	ADCY6, CLCF1, EDN1, HDAC9, IKBKE, IKBKG, IL15RA, IL1B, IL1R1, IL21R, IL36G, ITGA2B, ITGA3, ITGB4, ITGB5, MAP2K6, MAP3K13, MAPK11, MKNK2, MYC, PDE1B, PDE8B, PRKCB, PRKCG, TGFB2, TNFSF10, TNFSF13, TNFSF15
Macrophage Alternative Activation Signaling Pathway	4.97	0.0789	-0.258	ADORA2B, ARG1, CIITA, CREB3L4, IL10, IL1B, IL36G, MAPK11, MARCO, MYC, SOCS1, SOCS3, STAT1, TGFB2, THBS1
Interleukin-4 and Interleukin-13 signaling	4.74	0.1	0.302	HMOX1, IL10, IL1B, LAMA5, LCN2, MMP2, MYC, S1PR1, SOCS1, SOCS3, STAT1
OAS Antiviral Response	4.67	0.444	2	OAS1, OAS2, OAS3, RIGI
p38 MAPK Signaling	4.38	0.0917	0	CREB3L4, DDIT3, IL1B, IL1R1, IL36G, MAP2K6, MAPK11, MKNK2, MYC, STAT1, TGFB2
ISGylation Signaling Pathway	4.07	0.0926	1.897	EIF2AK2, IRF7, ITGA3, RIGI, STAT1, STAT2, TLR3, TLR8, UBA7, USP18
IL-12 Signaling and Production in Macrophages	4.05	0.0658	1.807	CCR5, DHX58, IKBKE, IKBKG, IL10, MAP2K6, MAPK11, PRKCB, PRKCG, RIGI, SOCS3, STAT1, TGFB2, THBS1, TLR3
STAT3 Pathway	3.92	0.0815	1.134	CISH, EGF, IL15RA, IL1B, IL1R1, IL21R, MAPK11, MYC, SOCS1, SOCS3, TGFB2
Role of PKR in Interferon Induction and Antiviral Response	3.89	0.0809	1.897	EIF2AK2, IKBKE, IKBKG, IL1B, MAP2K6, MAPK11, MARCO, RIGI, STAT1, STAT2, TLR3
Acute Phase Response Signaling	3.87	0.0703	0.632	CFB, HMOX1, HP, IKBKE, IKBKG, IL1B, IL1R1, IL36G, MAP2K6, MAPK11, Saa3, SOCS1, SOCS3
FXR/RXR Activation	3.82	0.0695	-0.277	CLCF1, DDIT3, FOXA1, GCLM, HMOX1, IL1B, IL36G, NQO1, SOCS3, TGFB2, TNFSF10, TNFSF13, TNFSF15
Death Receptor Signaling	3.76	0.0938	1.667	IKBKE, IKBKG, PARP10, PARP12, PARP14, PARP16, PARP9, TNFSF10, TNFSF15
HMGB1 Signaling	3.7	0.0719	0	CLCF1, IL1B, IL1R1, IL36G, MAP2K6, MAPK11, PLAT, RHOV, TGFB2, TNFSF10, TNFSF13, TNFSF15
Toll-like Receptor Signaling	3.67	0.103	0.816	EIF2AK2, IKBKG, IL1B, IL36G, MAP2K6, MAPK11, TLR3, TLR8
Role of JAK Family Kinases in IL-6-type Cytokine Signaling	3.64	0.101	1.414	CLCF1, IL10, MAPK11, MYC, SOCS1, SOCS3, STAT1, TGFB2
Adrenergic Receptor Signaling Pathway (Enhanced)	3.53	0.065	0.277	ADCY6, ATP6V0D2, CLCF1, GNAL, IL1B, IL36G, IL4I1, PRKCB, PRKCG, TGFB2, TNFSF10, TNFSF13, TNFSF15
PI3K/AKT Signaling	3.53	0.065	1.342	EPM2A, GDF15, IKBKE, IKBKG, IL15RA, IL1R1, IL21R, INPP5J, ITGA2B, ITGA3, ITGB4, ITGB5, SFN
Phagosome Formation	3.49	0.0417	-0.557	ADORA2B, CCR3, CCR5, CCRL2, CELSR3, CNR2, CX3CR1, GPR146, GPR183, HMOX1, ITGA2B, ITGA3, ITGB4, ITGB5,

				MAP2K6, MARCO, MYL2, P2RY12, PRKCB, PRKCG, PTGER1, PTGER2, S1PR1, SSTR5, SUCNR1, TLR3, TLR8, VIPR1, VIPR2
Coronavirus Pathogenesis Pathway	3.45	0.0637	-1.941	DDIT3, HDAC9, IL1B, IRF7, MAPK11, OAS1, Oas1b, OAS2, OAS3, RIGI, STAT1, STAT2, TLR3
Class A/1 (Rhodopsin-like receptors)	3.44	0.0536	-1.698	ADORA2B, APLN, CCR3, CCR5, CCRL2, CNR2, CX3CR1, CXCL10, CXCL16, EDN1, GPR183, P2RY12, PTGER1, PTGER2, S1PR1, SSTR5, SUCNR1
Neuroinflammation Signaling Pathway	3.44	0.0536	0.775	Aph1c, CREB3L4, CX3CR1, CXCL10, HMOX1, IKBKE, IKBKG, IL10, IL1B, IL1R1, IRF7, MAPK11, PRKCG, STAT1, TGFB2, TLR3, TLR8
Hepatic Fibrosis Signaling Pathway	3.31	0.048	0.243	CREB3L4, EDN1, IKBKE, IKBKG, IL1B, IL1R1, IL36G, ITGA2B, ITGA3, ITGB4, ITGB5, MAP2K6, MAPK11, MYC, MYL2, PRKCB, PRKCG, RHOV, SUCNR1, TGFB2
NFE2L2 Regulating Anti-oxidant/Detoxification Enzymes	3.26	0.211	2	GCLC, GCLM, HMOX1, NQO1
Breast Cancer Regulation by Stathmin1	3.14	0.0421	0.408	ADORA2B, ARHGEF4, CCR3, CCR5, CCRL2, CELSR3, CNR2, CREB3L4, CX3CR1, EGF, GPR146, GPR183, MMP2, P2RY12, PRKCB, PRKCG, PTGER1, PTGER2, S1PR1, SHC3, SSTR5, SUCNR1, TGFB2, VIPR1, VIPR2
Parkinson's Signaling Pathway	3.13	0.0521	1	ATP6V0D2, CLCF1, CNR2, DDIT3, IL1B, IL36G, IL4I1, MAPK11, NQO1, SLC40A1, TGFB2, TLR3, TLR8, TNFSF10, TNFSF13, TNFSF15
Neuregulin Signaling	3.12	0.0769	0.447	EGF, ERRFI1, ITGA2B, ITGA3, ITGB4, ITGB5, MYC, PRKCB, PRKCG
Hepatic Cholestasis	3.09	0.0583	0.832	ADCY6, CLCF1, IKBKE, IKBKG, IL1B, IL1R1, IL36G, PRKCB, PRKCG, TGFB2, TNFSF10, TNFSF13, TNFSF15
MSP-RON Signaling in Macrophages Pathway	3.07	0.0756	-1.667	ARG1, CIITA, CREB3L4, IKBKE, IKBKG, IL10, SOCS1, SOCS3, STAT1
CREB Signaling in Neurons	3.01	0.0412	0.209	ADCY6, ADORA2B, CCR3, CCR5, CCRL2, CELSR3, CNR2, CREB3L4, CX3CR1, EGF, GNAL, GPR146, GPR183, P2RY12, PRKCB, PRKCG, PTGER1, PTGER2, S1PR1, SHC3, SSTR5, SUCNR1, TGFB2, VIPR1, VIPR2
Endocannabinoid Cancer Inhibition Pathway	2.99	0.068	0	ADCY6, CASP4, CNR2, CREB3L4, DDIT3, GNAL, MAP2K6, MMP2, MYC, SMPD3
NLR signaling pathways	2.98	0.107	1.633	CASP4, HMOX1, IKBKG, MAP2K6, MAPK11, PANX1
TREM1 Signaling	2.96	0.0909	-1.134	CD83, CIITA, IL10, IL1B, NLRC5, TLR3, TLR8
NF-κB Activation by Viruses	2.93	0.0897	1.633	CCR5, EIF2AK2, IKBKE, IKBKG, ITGA3, PRKCB, PRKCG
Type II Diabetes Mellitus Signaling	2.86	0.0654	1	ACSBG1, IKBKE, IKBKG, MAFA, PRKCB, PRKCG, SLC27A3, SMPD3, SOCS1, SOCS3
cAMP-mediated Signaling	2.86	0.0551	0.277	ADCY6, ADORA2B, CNGA3, CNR2, CREB3L4, GNAL, P2RY12, PDE1B, PDE8B, PTGER2, S1PR1, VIPR1, VIPR2
Growth Hormone Receptor Signaling	2.86	0.167	-1	CISH, SOCS1, SOCS3, STAT1

IL-27 Signaling Pathway	2.85	0.0703	0.333	ENTPD1, GADD45G, IL10, IL1R1, MAPK11, SOCS3, STAT1, TLR3, TLR8
Retinoic acid Mediated Apoptosis Signaling	2.83	0.1	2.449	PARP10, PARP12, PARP14, PARP16, PARP9, TNFSF10
GADD45 Signaling	2.83	0.1	1.633	GADD45G, IL1B, MAP2K6, MAPK11, MYC, TGFB2
IL-6 Signaling	2.82	0.0698	0.707	IKBKE, IKBKG, IL1B, IL1R1, IL36G, MAP2K6, MAPK11, SOCS1, SOCS3
Extracellular Matrix Organization	2.78	0.0755	0.707	AGRN, CEACAM1, ITGA2B, ITGA3, ITGB4, ITGB5, LAMA5, TGFB2
IL-17 Signaling	2.73	0.0588	0.905	CLCF1, IL1B, IL36G, LCN2, MAP2K6, MAPK11, MMP2, TGFB2, TNFSF10, TNFSF13, TNFSF15
Role of Osteoclasts in Rheumatoid Arthritis Signaling Pathway	2.68	0.0487	1.387	ADAM22, Adam4/Gm4787, COL15A1, COL23A1, CREB3L4, IKBKE, IKBKG, IL10, IL1B, IL1R1, MAP2K6, MAPK11, MMP2, RHOV, SHC3
Fatty acyl-CoA biosynthesis	2.66	0.148	0	ACSBG1, HACD4, SCD, SLC27A3
Sleep REM Signaling Pathway	2.66	0.0721	0	CLCF1, CREB3L4, IL1B, IL36G, TGFB2, TNFSF10, TNFSF13, TNFSF15
Regulation of the Epithelial Mesenchymal Transition by Growth Factors Pathway	2.64	0.0573	1.265	EGF, IKBKE, IKBKG, MAP2K6, MAPK11, MMP2, SHC3, TGFB2, TNFSF10, TNFSF13, TNFSF15
Interleukin-10 Signaling	2.64	0.111	-2.236	CCR5, CXCL10, IL10, IL1B, IL1R1
Endothelin-1 Signaling	2.6	0.0567	0.632	ADCY6, CASP4, EDN1, GNAL, HMOX1, MAPK11, MYC, PRKCB, PRKCG, PTGER2, SHC3
Cytosolic Sensors of Pathogen-associated DNA	2.59	0.109	2.236	IFI16, IKBKG, IRF7, TLR3, ZBP1
Role of RIG1-like Receptors in Antiviral Innate Immunity	2.59	0.109	1.342	DHX58, IKBKE, IKBKG, IRF7, RIGI
G alpha (i) Signaling Events	2.52	0.0504	-0.277	ADCY6, APLN, CCR3, CCR5, CNR2, CX3CR1, CXCL10, CXCL16, GPR183, P2RY12, RGS11, SSTR5, SUCNR1
Ribonucleotide Reductase Signaling Pathway	2.51	0.0585	2.53	CREB3L4, MAPK11, MMP2, MYC, PARP10, PARP12, PARP14, PARP16, PARP9, THBS1
Prolactin Signaling	2.44	0.0737	0.816	ABCC9, MYC, PRKCB, PRKCG, SOCS1, SOCS3, STAT1
Erythropoietin Signaling Pathway	2.42	0.0568	-0.333	CLCF1, IL1B, IL36G, PRKCB, PRKCG, SHC3, TGFB2, TNFSF10, TNFSF13, TNFSF15
IL-1 Signaling	2.42	0.0729	0	ADCY6, GNAL, IKBKE, IKBKG, IL1R1, MAP2K6, MAPK11
RAR Activation	2.4	0.042	1.414	ADCY6, CLCF1, CNGA3, CREB3L4, EGF, IL1B, IL36G, MAPK11, MMP2, PDE1B, PDE8B, RDH5, RHOV, SOCS3, TGFB2, TNFSF10, TNFSF13, TNFSF15
Toll Like Receptor 3 (TLR3) Cascade	2.38	0.125	2	IKBKE, IKBKG, IRF7, TLR3

Role of Macrophages, Fibroblasts and Endothelial Cells in Rheumatoid Arthritis	2.37	0.045	0.535	CREB3L4, IKBKE, IKBKG, IL10, IL1B, IL1R1, IL36G, MAP2K6, MYC, PRKCB, PRKCG, SOCS1, SOCS3, TLR3, TLR8
Serotonin Receptor Signaling	2.34	0.0405	0.688	ADCY6, CREB3L4, EDN1, FBP2, GNAL, HMOX1, IL1B, IL4I1, ITGA2B, MAP2K6, MAPK11, MMP2, MYC, MYL2, PRKCB, PRKCG, RHOV, SLC9A5, TGFB2
Activin Inhibin Signaling Pathway	2.32	0.0521	0.905	IKBKE, IKBKG, IL1B, IL1R1, IL36G, MAP2K6, MAPK11, MMP2, TGFB2, TLR3, TLR8
Gαs Signaling	2.32	0.0635	0	ADCY6, ADORA2B, CNGA3, CREB3L4, GNAL, PTGER2, VIPR1, VIPR2
Cellular Effects of Sildenafil (Viagra)	2.29	0.0363	1	ADCY6, ADORA2B, CCR3, CCR5, CCRL2, CELSR3, CNGA3, CNR2, CREB3L4, CX3CR1, GPR146, GPR183, MAP2K6, MAPK11, MYL2, P2RY12, PTGER1, PTGER2, S1PR1, SSTR5, STAR, SUCNR1, TGFB2, VIPR1, VIPR2
Interleukin-1 Family Signaling	2.26	0.062	0.707	IKBKG, IL18BP, IL1B, IL1R1, IL36G, MAP2K6, PTPN14, PTPN5
VDR/RXR Activation	2.25	0.0769	0	CXCL10, HSD17B2, IGFBP5, PRKCB, PRKCG, TGFB2
DDX58/IFIH1-mediated Induction of Interferon-alpha/beta	2.22	0.0759	2.449	IKBKE, IKBKG, IRF7, NLRC5, RIGI, UBA7
S100 Family Signaling Pathway	2.22	0.035	-0.577	ADORA2B, CCR3, CCR5, CCRL2, CELSR3, CNR2, CREB3L4, CX3CR1, EGF, GPR146, GPR183, IL1B, MAP2K6, MAPK11, MMP2, P2RY12, PLAT, PRKCB, PRKCG, PTGER1, PTGER2, S1PR1, SSTR5, SUCNR1, TGFB2, VIPR1, VIPR2
IL-17A Signaling in Fibroblasts	2.2	0.075	0.816	IKBKE, IKBKG, IL1B, LCN2, MAPK11, PRKCB
BBSome Signaling Pathway	2.16	0.0389	-0.943	ADORA2B, CBX8, CCR3, CCR5, CCRL2, CELSR3, CNR2, CX3CR1, GPR146, GPR183, MAPK11, P2RY12, PTGER1, PTGER2, S1PR1, SSTR5, SUCNR1, VIPR1, VIPR2
PPARα/RXRα Activation	2.13	0.0515	-0.333	ADCY6, HELZ2, IKBKE, IKBKG, IL1B, IL1R1, ITGB5, MAP2K6, PRKCB, TGFB2
Integrin Cell Surface Interactions	2.07	0.0706	0.816	AGRN, COL23A1, ITGA2B, ITGA3, ITGB5, THBS1
LPS-stimulated MAPK Signaling	2.07	0.0706	0.447	IKBKE, IKBKG, MAP2K6, MAPK11, PRKCB, PRKCG
Systemic Lupus Erythematosus in B Cell Signaling Pathway	2.03	0.0346	0.816	CD79B, CLCF1, IFIT2, IFIT3, Ighv1-58, IL10, IL1B, IL36G, INPP5J, IRF7, ISG20, LILRB4, MYC, PRKCB, PRKCG, RASGRP3, SHC3, STAT1, STAT2, TGFB2, TLR3, TLR8, TNFSF10, TNFSF13, TNFSF15
CDX Gastrointestinal Cancer Signaling Pathway	2.01	0.0495	-0.632	CLCF1, IKBKE, IKBKG, IL1B, IL36G, MAPK11, TGFB2, TNFSF10, TNFSF13, TNFSF15
Ephrin Receptor Signaling	2.01	0.0495	2.236	CREB3L4, EGF, EPHB3, GNAL, ITGA2B, ITGA3, ITGB4, ITGB5, NCK2, SH2D3C
Xenobiotic Metabolism General Signaling Pathway	2	0.0559	1.414	GCLC, HMOX1, MAP2K6, MAP3K13, MAPK11, NQO1, PRKCB, PRKCG
IL-13 Signaling Pathway	1.97	0.0603	1.134	ARG1, IL10, MAPK11, SOCS1, SOCS3, STAT1, TGFB2

Immunogenic Cell Death Signaling Pathway	1.96	0.0667	0.816	CXCL10, DDIT3, GZMM, IL1B, PANX1, TLR3
PAK Signaling	1.96	0.0598	1	EPHB3, ITGA2B, ITGA3, ITGB4, ITGB5, MYL2, NCK2
Colorectal Cancer Metastasis Signaling	1.94	0.0443	1.265	ADCY6, EGF, GNAL, MMP2, MYC, PTGER1, PTGER2, RHOV, STAT1, TGFB2, TLR3, TLR8
IL-8 Signaling	1.9	0.0476	1.414	GNAL, HMOX1, IKBKE, IKBKG, MMP2, MYL2, PRKCB, PRKCG, RAB11FIP2, RHOV
ERBB Signaling	1.89	0.0645	0.447	EGF, MAP2K6, MAPK11, NCK2, PRKCB, PRKCG
Th1 Pathway	1.86	0.0574	-0.378	Aph1c, CCR5, IL10, MAP2K6, SOCS1, SOCS3, STAT1
Role of JAK1 and JAK3 in γc Cytokine Signaling	1.85	0.0725	2.236	IL15RA, IL21R, SOCS1, SOCS3, STAT1
Eicosanoid Signaling	1.84	0.0429	0.577	ABCC9, ADCY6, CREB3L4, GNAL, IL10, MAPK11, MMP2, MYC, PRKCB, PRKCG, PTGER1, PTGER2
ERK/MAPK Signaling	1.83	0.0465	0.378	CREB3L4, ITGA2B, ITGA3, ITGB4, ITGB5, MKNK2, MYC, PRKCB, PRKCG, STAT1
Docosahexaenoic Acid (DHA) Signaling	1.8	0.044	1.508	ADCY6, Aph1c, CREB3L4, EGF, HMOX1, IL1B, OSGIN1, PRKCB, PRKCG, SYT3, TGFB2
ISG15 Antiviral Mechanism	1.8	0.0704	2.236	EIF2AK2, IFIT1, RIGI, STAT1, UBA7
Growth Hormone Signaling	1.8	0.0704	1.342	PRKCB, PRKCG, SOCS1, SOCS3, STAT1
FAK Signaling	1.79	0.0309	0.707	ADORA2B, CCR3, CCR5, CCRL2, CELSR3, CNR2, CX3CR1, EGF, GPR146, GPR183, IL15RA, IL1R1, IL21R, ITGA2B, ITGA3, ITGB4, ITGB5, MAPK11, MMP2, MYC, NCK2, P2RY12, PTGER1, PTGER2, S1PR1, SOCS1, SOCS3, SSTR5, SUCNR1, TGFB2, VIPR1, VIPR2
RHOGDI Signaling	1.77	0.0455	-1.342	ARHGEF4, CDH24, GNAL, GRIP1, ITGA2B, ITGA3, ITGB4, ITGB5, MYL2, RHOV
Apelin Cardiomyocyte Signaling Pathway	1.77	0.0606	0	APLN, MAPK11, MYL2, PRKCB, PRKCG, SLC9A5
PFKFB4 Signaling Pathway	1.73	0.0816	1	CREB3L4, FBP2, MAP2K6, TGFB2
Thrombin Signaling	1.71	0.0444	0.816	ADCY6, ARHGEF37, ARHGEF4, EGF, GNAL, MAPK11, MYL2, PRKCB, PRKCG, RHOV
Caveolar-mediated Endocytosis Signaling	1.7	0.0667	0.447	EGF, ITGA2B, ITGA3, ITGB4, ITGB5
Response to Elevated Platelet Cytosolic Ca2+	1.69	0.053	1.134	EGF, GTPBP2, ITGA2B, PRKCB, PRKCG, TGFB2, THBS1
HGF Signaling	1.69	0.053	1	ITGA2B, ITGA3, ITGB4, ITGB5, MAP3K13, PRKCB, PRKCG
P2Y Purinergic Receptor Signaling Pathway	1.68	0.0526	0.816	ADCY6, CREB3L4, ITGA2B, MYC, P2RY12, PRKCB, PRKCG
Role of MAPK Signaling in Inhibiting the Pathogenesis of Influenza	1.62	0.0633	-0.447	CXCL10, EIF2AK2, IL1B, MAP2K6, MAPK11
Reelin Signaling in Neurons	1.6	0.0507	1.633	ARHGEF37, ARHGEF4, CNR2, ITGA3, LRP8, MAP2K6, MAPK8IP3

Protein Kinase A Signaling	1.6	0.0365	1.155	ADCY6, CNGA3, CREB3L4, EPM2A, MYL2, PDE1B, PDE8B, PRKCB, PRKCG, PTPN14, PTPN5, PTPRF, Ptprv, SFN, TGFB2
Gαq Signaling	1.59	0.0471	1.633	EPM2A, GNAL, HMOX1, IKBKE, IKBKG, PRKCB, PRKCG, RHOV
Chemokine Signaling	1.58	0.0617	-2	CCR3, CCR5, MAPK11, MYL2, PRKCB
JAK/STAT Signaling	1.56	0.061	2.236	CISH, SOCS1, SOCS3, STAT1, STAT2
O-linked Glycosylation	1.55	0.0541	-0.816	ADAMTS6, B3GNT6, GALNT3, SEMA5B, ST6GAL1, THBS1
Role of Tissue Factor in Cancer	1.53	0.0435	1.667	EGF, IKBKE, IKBKG, IL1B, ITGA3, MAP2K6, MAPK11, MMP2, TGFB2
TNF Signaling	1.52	0.0702	0	IKBKE, IKBKG, OTUD1, SMPD3
CD27 Signaling in Lymphocytes	1.52	0.0702	1	IKBKE, IKBKG, MAP2K6, MAP3K13
HIF1α Signaling	1.51	0.0431	0.333	EDN1, EGF, HMOX1, MAP2K6, MKNK2, MMP2, PRKCB, PRKCG, TGFB2
Role of Osteoblasts in Rheumatoid Arthritis Signaling Pathway	1.5	0.041	1.265	CLCF1, IL1B, IL36G, MMP2, STAT1, STAT2, TGFB2, TNFSF10, TNFSF13, TNFSF15
G alpha (s) Signaling Events	1.5	0.0483	0.378	ADCY6, ADORA2B, PDE1B, PDE8B, PTGER2, VIPR1, VIPR2
Signaling by PDGF	1.49	0.069	0	NCK2, PLAT, STAT1, THBS1
Iron uptake and Transport	1.49	0.069	1	ATP6V0D2, HMOX1, LCN2, SLC40A1
Oxidative Stress Induced Senescence	1.48	0.0581	1	CBX4, CBX8, MAP2K6, MAPK11, mir-24 (includes others)
Tumor Microenvironment Pathway	1.47	0.0447	-0.707	ARG1, EGF, IDO2, IL10, IL1B, MMP2, MYC, TGFB2
PDGF Signaling	1.46	0.0575	-0.447	EIF2AK2, INPP5J, MYC, PRKCB, STAT1
CLEAR Signaling Pathway	1.44	0.0386	-0.302	ATP6V0D2, CREB3L4, EGF, MAPK11, PRKCB, PRKCG, SFN, SGSH, TGFB2, TLR3, TLR8
Semaphorin Neuronal Repulsive Signaling Pathway	1.43	0.0467	0	ITGA2B, ITGA3, ITGB4, ITGB5, MAP2K6, MYL2, SEMA4D
Cholecystokinin/Gastrin-mediated Signaling	1.42	0.0504	0	IL1B, IL36G, MAP2K6, PRKCB, PRKCG, RHOV
NAD Signaling Pathway	1.42	0.0464	2.646	EGF, PARP10, PARP12, PARP14, PARP16, PARP9, TGFB2
PTEN Signaling	1.42	0.0464	-2	IKBKE, IKBKG, INPP5J, ITGA2B, ITGA3, ITGB4, ITGB5
NGF Signaling	1.41	0.05	1.342	CREB3L4, IKBKE, IKBKG, MAP3K13, SHC3, SMPD3
Corticotropin Releasing Hormone Signaling	1.4	0.0461	0.816	ADCY6, CREB3L4, Gucy2g, MAPK11, NR4A1, PRKCB, PRKCG
Glutaminergic Receptor Signaling Pathway (Enhanced)	1.4	0.0369	2.309	ADCY6, CREB3L4, DGKI, GNAL, HMOX1, IKBKE, IKBKG, KCNK10, MAPK11, PRKCB, PRKCG, TGFB2
IL-33 Signaling Pathway	1.4	0.0432	1.414	CASP4, CREB3L4, IKBKE, IKBKG, IL1B, IL36G, MAPK11, MMP2
RANK Signaling in Osteoclasts	1.39	0.0549	1	IKBKE, IKBKG, MAP2K6, MAP3K13, MAPK11
Myelination Signaling Pathway	1.39	0.0367	-0.577	ADAM22, Aph1c, CREB3L4, EGR2, HDAC9, ITGA3, ITGB4, ITGB5, LAMA5, PLP1, SCD, XAF1
Thrombopoietin Signaling	1.38	0.0635	0	MYC, PRKCB, PRKCG, STAT1

Necroptosis Signaling Pathway	1.37	0.0452	2.646	EIF2AK2, IKBKG, STAT1, STAT2, TLR3, TNFSF10, ZBP1
GNRH Signaling	1.34	0.0419	1.134	ADCY6, CREB3L4, MAP2K6, MAP3K13, MAPK11, MMP2, PRKCB, PRKCG
Production of Nitric Oxide and Reactive Oxygen Species in Macrophages	1.34	0.0419	1.89	IKBKE, IKBKG, MAP3K13, MAPK11, PRKCB, PRKCG, RHOV, STAT1
HER-2 Signaling in Breast Cancer	1.32	0.0396	0.333	EGF, IKBKE, IKBKG, ITGB4, ITGB5, MMP2, MYC, PRKCB, PRKCG
GP6 Signaling Pathway	1.31	0.0472	0.816	COL15A1, COL23A1, ITGA2B, LAMA5, PRKCB, PRKCG
Endocannabinoid Developing Neuron Pathway	1.31	0.0472	0.447	ADCY6, CNR2, CREB3L4, GNAL, MAP2K6, MAPK11
Acetylcholine Receptor Signaling Pathway	1.3	0.0412	1.414	ADCY6, Aph1c, CASP4, CREB3L4, GNAL, HMOX1, PRKCB, PRKCG
Pulmonary Healing Signaling Pathway	1.25	0.0402	0	EGF, MAPK11, MMP2, MYC, PRKCB, PRKCG, TGFB2, THBS1
Adrenomedullin Signaling Pathway	1.25	0.0402	0.378	ADCY6, Gucy2g, IL1B, IL36G, MAP2K6, MAPK11, MMP2, SHC3
Osteoarthritis Pathway	1.23	0.0381	-0.816	CASP4, CCN4, CREB3L4, IL1B, IL1R1, ITGA2B, ITGA3, ITGB4, ITGB5
ILK Signaling	1.23	0.0398	-0.707	CREB3L4, ITGB4, ITGB5, MAP2K6, MYC, MYL2, NCK2, RHOV
NRF2-mediated Oxidative Stress Response	1.23	0.038	1.134	AOX1, CYP2S1, GCLC, GCLM, HMOX1, MAP2K6, NQO1, PRKCB, PRKCG
Th2 Pathway	1.18	0.0438	-0.816	Aph1c, CCR3, CCR5, IL10, S1PR1, SOCS3
ERK5 Signaling	1.17	0.0541	1	CREB3L4, EGF, MYC, SFN
Gαi Signaling	1.14	0.0429	-1	ADCY6, CNR2, GNAL, P2RY12, S1PR1, SHC3
PPAR Signaling	1.14	0.0467	-0.447	IKBKE, IKBKG, IL1B, IL1R1, IL36G
Pancreatic Secretion Signaling Pathway	1.13	0.0363	0.333	ADCY6, ADORA2B, ARHGEF4, EGF, PRKCB, PRKCG, RASGRP3, VIPR1, VIPR2
Neutrophil Extracellular Trap Signaling Pathway	1.13	0.0325	0.277	CASP4, CCR5, COL15A1, COL23A1, IL10, IL1B, ITGA3, MAP2K6, MAPK11, MMP2, PRKCB, PRKCG, TLR8
Pulmonary Fibrosis Idiopathic Signaling Pathway	1.1	0.0337	-0.302	CCN4, COL15A1, COL23A1, EDN1, EPHB3, IL1B, MAP2K6, MAPK11, MMP2, TGFB2, THBS1
Sleep NREM Signaling Pathway	1.04	0.0435	-1.342	ADCY6, ADORA2B, CREB3L4, MAP2K6, SFN
Role of NFAT in Cardiac Hypertrophy	1.02	0.0357	0.447	ADCY6, HDAC9, MAP2K6, MAPK11, PRKCB, PRKCG, SHC3, TGFB2
Fc Epsilon RI Signaling	1	0.0424	0	INPP5J, MAP2K6, MAPK11, PRKCB, PRKCG
Factors Promoting Cardiogenesis in Vertebrates	1	0.0392	0	CREB3L4, MYC, MYL2, PRKCB, PRKCG, TGFB2
Sphingosine-1-phosphate Signaling	0.981	0.0417	-1.342	ADCY6, CASP4, RHOV, S1PR1, SMPD3
Signaling by Rho Family GTPases	0.979	0.0337	1.342	ARHGEF4, CDH24, GNAL, ITGA2B, ITGA3, ITGB4, ITGB5, MYL2, RHOV

LXR/RXR Activation	0.948	0.0407	0	IL1B, IL1R1, IL36G, SCD, TLR3
Opioid Signaling	0.932	0.0444	2	ADCY6, GNAL, PDE1B, PRKCG
Sertoli Cell-Germ Cell Junction Signaling Pathway (Enhanced)	0.925	0.0339	0.707	IL1R1, ITGA3, LAMA5, MAP2K6, MAP3K13, MAPK11, MMP2, TGFB2
Insulin Secretion Signaling Pathway	0.921	0.0327	1.667	ABCC9, ADCY6, CREB3L4, MAFA, MAPK11, PRKCB, PRKCG, STAT1, STAT2
Opioid Signaling Pathway	0.887	0.0321	-0.333	ADCY6, CREB3L4, GNAL, MAP2K6, MYC, PDE1B, PRKCB, PRKCG, RGS11
EPH-Ephrin Signaling	0.882	0.0426	1	Aph1c, EPHB3, MMP2, NCK2
Fcγ Receptor-mediated Phagocytosis in Macrophages and Monocytes	0.882	0.0426	1	HMOX1, NCK2, PRKCB, PRKCG
Oxytocin Signaling Pathway	0.874	0.0319	1	ABCC9, CREB3L4, GNAL, Gucy2g, MAPK11, MYL2, PRKCB, PRKCG, SHC3
Sertoli Cell-Sertoli Cell Junction Signaling	0.873	0.0329	0.707	CDH24, CGN, CREB3L4, IL1R1, ITGA3, LAMA5, MAP3K13, MAPK11
CXCR4 Signaling	0.863	0.0357	-0.447	ADCY6, GNAL, MYL2, PRKCB, PRKCG, RHOV
Gap Junction Signaling	0.854	0.0308	0	ADCY6, CREB3L4, EGF, Gucy2g, IL1B, MYC, P2RY12, PRKCB, PRKCG, PTGER2
Salvage Pathways of Pyrimidine Ribonucleotides	0.846	0.0412	0	CMPK2, EIF2AK2, MAP2K6, UPP2
Estrogen Receptor Signaling	0.838	0.0293	1.155	ADCY6, CREB3L4, EGF, FOXA1, GNAL, MDK, MMP2, MYC, MYL2, PRKCB, PRKCG, SHC3
Integrin Signaling	0.817	0.033	0.378	ITGA2B, ITGA3, ITGB4, ITGB5, MYL2, NCK2, RHOV
p75 NTR Receptor-mediated Signaling	0.813	0.04	1	Aph1c, ARHGEF37, ARHGEF4, MCF2L
ABC-family Proteins Mediated Transport	0.802	0.0396	-1	ABCA6, ABCA8, ABCC9, ABCD2
Ephrin A Signaling	0.789	0.036	-1.342	Aph1c, EGF, MYL2, NCK2, TGFB2
Role of Chondrocytes in Rheumatoid Arthritis Signaling Pathway	0.771	0.0355	0.447	IL1B, IL1R1, IL36G, MAPK11, MMP2
Apelin Endothelial Signaling Pathway	0.771	0.0355	-0.447	ADCY6, APLN, GNAL, PRKCB, PRKCG
Cardiac Hypertrophy Signaling	0.754	0.0307	0.816	ADCY6, GNAL, MAP2K6, MAP3K13, MAPK11, MYL2, RHOV, TGFB2
MicroRNA Biogenesis Signaling Pathway	0.714	0.0321	0.816	ADAR, EGF, HMOX1, MAPK11, MYC, TGFB2
Dilated Cardiomyopathy Signaling Pathway	0.696	0.0333	1.342	ABCC9, ADCY6, MAPK11, MYL2, RBM20
Leukocyte Extravasation Signaling	0.673	0.0311	1.342	ITGA3, MAPK11, MMP2, PRKCB, PRKCG, TIMP2

3-phosphoinositide Degradation	0.666	0.0309	-2.236	DUSP14, INPP5J, PALD1, PANK4, PTPRF, SOCS3
Superpathway of Inositol Phosphate Compounds	0.653	0.0295	-1.633	DUSP14, INPP5J, IP6K3, PALD1, PANK4, PTPRF, SOCS3
Orexin Signaling Pathway	0.647	0.0294	1.134	ADCY6, ATP6V0D2, IL1B, MAPK11, PRKCB, PRKCG, STAR
Virus Entry via Endocytic Pathways	0.64	0.0339	-1	ITGB4, ITGB5, PRKCB, PRKCG
Oxytocin in Brain Signaling Pathway	0.634	0.0302	0.816	CREB3L4, DDIT3, GNAL, IL1B, PRKCB, PRKCG
ID1 Signaling Pathway	0.621	0.0299	0	BHLHE41, EGF, MAPK11, MMP2, MYC, TGFB2
Inhibition of ARE-Mediated mRNA Degradation Pathway	0.602	0.0307	0.447	MAPK11, SFN, TNFSF10, TNFSF13, TNFSF15
Regulation of Insulin-like Growth Factor (IGF) transport and uptake by IGFBPs	0.591	0.0323	0	FAM20A, IGFBP5, MMP2, STC2
L1CAM interactions	0.584	0.032	1	DLG3, ITGA2B, LAMA5, NRCAM
Senescence Pathway	0.55	0.0268	0.707	CBX8, GADD45G, IKBKE, IKBKG, MAP2K6, Saa3, TGFB2, YPEL3
Neutrophil degranulation	0.545	0.0252	-0.577	ARG1, CEACAM1, CRISPLD2, HP, LCN2, OLR1, PTX3, SERPINB12, SIRPB1, SLPI, TARM1, TIMP2
Gα12/13 Signaling	0.526	0.0301	0	CDH24, IKBKE, IKBKG, MYL2
Autism Signaling Pathway	0.511	0.026	0.707	CLCF1, IL1B, IL36G, MTHFR, TGFB2, TNFSF10, TNFSF13, TNFSF15
Hereditary Breast Cancer Signaling	0.494	0.029	0	EGF, GADD45G, SFN, TGFB2
D-myo-inositol (1, 4, 5, 6)-Tetrakisphosphate Biosynthesis	0.481	0.0273	-2	DUSP14, PALD1, PANK4, PTPRF, SOCS3
D-myo-inositol (3, 4, 5, 6)-tetrakisphosphate Biosynthesis	0.481	0.0273	-2	DUSP14, PALD1, PANK4, PTPRF, SOCS3
Synaptogenesis Signaling Pathway	0.481	0.0254	1.414	ADCY6, CDH24, CREB3L4, EPHB3, LRP8, SHC3, SYT3, THBS1
MSP-RON Signaling in Cancer Cells Pathway	0.481	0.0286	1	CREB3L4, ITGB4, MYC, SFN
Chronic Myeloid Leukemia Signaling	0.45	0.0252	0.378	ADAR, HDAC9, IKBKE, IKBKG, MYC, TGFB2, TNFSF10
WNT/SHH Axonal Guidance Signaling Pathway	0.429	0.0268	1	ADCY6, CELSR3, SEMA3C, SFN
Huntington's Disease Signaling	0.426	0.0246	0	CASP4, CREB3L4, EGF, HDAC9, PRKCB, PRKCG, SHC3
Transcriptional regulation by RUNX1	0.413	0.0263	0	CBX4, CBX8, PRKCB, SOCS3
D-myo-inositol-5-phosphate Metabolism	0.403	0.0251	-2	DUSP14, PALD1, PANK4, PTPRF, SOCS3
Human Embryonic Stem Cell Pluripotency	0.394	0.0249	-0.447	MAP2K6, MYC, PRKCB, PRKCG, TGFB2
Gustation Pathway	0.385	0.0246	0.447	ABCC9, ADCY6, P2RX6, P2RY12, PANX1

Histone Modification Signaling Pathway	0.385	0.0237	1.134	CBX4, DNMT3L, MMP2, MYC, PRDM9, PRKCB, SFN
Epithelial Adherens Junction Signaling	0.382	0.0253	0	EGF, NANOS1, SFN, TGFB2
HEY1 Signaling Pathway	0.368	0.0248	0	Aph1c, ITGA2B, MMP2, TGFB2
3-Phosphoinositide Biosynthesis	0.36	0.0239	-2	DUSP14, PALD1, PANK4, PTPRF, SOCS3
Cell surface interactions at the vascular wall	0.352	0.0237	-0.447	CD2, CEACAM1, ITGA3, OLR1, PROCR
RAF/MAP kinase cascade	0.337	0.0229	0.816	DLG3, EGF, ITGA2B, KSR2, RASGRP3, SHC3
Deubiquitination	0.334	0.0228	0.816	H2AC1, IKBKG, MYC, RIGI, USP11, USP18
Autophagy	0.329	0.023	0.447	CREB3L4, DDIT3, EGF, MYC, TGFB2
Signaling by the B Cell Receptor (BCR)	0.328	0.0235	-1	CD79B, IKBKG, PRKCB, RASGRP3
Nuclear Cytoskeleton Signaling Pathway	0.318	0.0227	0.447	CDH24, ITGA2B, ITGA3, ITGB4, ITGB5
Netrin Signaling	0.316	0.0231	0	ADORA2B, NCK2, PRKCB, PRKCG
Protein Sorting Signaling Pathway	0.296	0.0225	2	ADCY6, HMOX1, SEC31B, SNX32
Class I MHC Mediated Antigen Processing and Presentation	0.287	0.0213	0.707	ASB4, HERC6, IKBKG, RNF213, SOCS1, SOCS3, TAP1, UBA7
Ion Channel Transport	0.278	0.0219	1	ATP6V0D2, CLCN1, MCOLN2, TRPM3
Generic Transcription Pathway	0.276	0.021	-0.333	HDAC9, NR4A1, ZNF112, ZNF205, ZNF442, ZNF558, ZNF605, ZNF667, ZNF703
Dopamine-DARPP32 Feedback in cAMP Signaling	0.268	0.0215	1	ADCY6, CREB3L4, PRKCB, PRKCG
Synaptic Long Term Depression	0	0.0202	1	GNAL, Gucy2g, PRKCB, PRKCG
Chaperone Mediated Autophagy Signaling Pathway	0	0.00787	0.447	ATP6V0D2, IL1B, MAPK11, MMP2, MYC
NFKBIE Signaling Pathway	0	0.0111	0.447	IKBKE, IKBKG, TNFSF10, TNFSF13, TNFSF15
RHO GTPase Cycle	0	0.02	1.667	ARHGAP24, ARHGEF4, BAIAP2L1, MCF2L, NCK2, PKN3, RHOV, RHPN2, ZNF512B
Role of NFAT in Regulation of the Immune Response	0	0.00484	1	CD79B, GNAL, Ighv1-58, IKBKE, IKBKG
CTLA4 Signaling in Cytotoxic T Lymphocytes	0	0.0132	-0.707	H2-K2/H2-Q9, H2-M2, H2-T24, HMOX1, IDO2, ITGA3, MAPK11, TGFB2
Dendritic Cell Maturation	0	0.0185	0.905	CD83, CREB3L4, IKBKE, IKBKG, IL10, IL1B, IL36G, MAPK11, STAT1, STAT2, TLR3
Type I Diabetes Mellitus Signaling	0	0.0177	0.707	IKBKE, IKBKG, IL1B, IL1R1, MAP2K6, MAPK11, SOCS1, SOCS3, STAT1
mTOR Signaling	0	0.0187	1	HMOX1, PRKCB, PRKCG, RHOV
Communication between Innate and Adaptive Immune Cells	0	0.0107	-1.265	CD79B, CD83, CXCL10, Ighv1-58, IL10, IL1B, IL36G, TLR3, TLR8, TNFSF13

Phospholipase C Signaling	0	0.0134	0.333	ADCY6, ARHGEF4, CD79B, CREB3L4, HDAC9, HMOX1, Ighv1-58, ITGA2B, ITGA3, ITGB4, ITGB5, MYL2, PRKCB, PRKCG, RHOV
NUR77 Signaling in T Lymphocytes	0	0.0156	0.447	H2-K2/H2-Q9, H2-M2, H2-T24, HDAC9, NR4A1, PRKCB, PRKCG, TNFSF10
TEC Kinase Signaling	0	0.019	1.633	GNAL, ITGA2B, ITGA3, ITGB4, ITGB5, PRKCB, PRKCG, RHOV, STAT1, STAT2, TNFSF10
IL-4 Signaling	0	0.0121	1.134	ARG1, COL15A1, COL23A1, CREB3L4, MAPK11, SOCS1, TGFB2
B Cell Receptor Signaling	0	0.0158	0.707	CD79B, CREB3L4, Ighv1-58, IKBKE, IKBKG, INPP5J, MAP2K6, MAP3K13, MAPK11, PRKCB
NF-κB Signaling	0	0.0175	0	EGF, EIF2AK2, IKBKG, IL1B, IL1R1, IL36G, MAP2K6, PRKCB, TLR3, TLR8
T Cell Receptor Signaling	0	0.0146	1.667	H2-K2/H2-Q9, H2-M2, H2-T24, IKBKE, IKBKG, ITGA3, MAP2K6, MAPK11, SHC3
Th17 Activation Pathway	0	0.0124	0	IL10, IL1B, IL1R1, IL21R, PTGER2, SOCS3
Systemic Lupus Erythematosus in T Cell Signaling Pathway	0	0.0124	0.707	CASP4, CREB3L4, H2-K2/H2-Q9, H2-M2, H2-T24, IL10, MAP2K6, RHOV
Xenobiotic Metabolism CAR Signaling Pathway	0	0.0206	1	GRIP1, MAP2K6, PRKCB, PRKCG
Xenobiotic Metabolism PXR Signaling Pathway	0	0.0205	1	GRIP1, IL4I1, PRKCB, PRKCG
