# Supplementary material for: Atractylodes lancea (Thunb.) DC. [Asteraceae] Rhizome-Derived Exosome-like Nanoparticles Suppress Lipopolysaccharide-Induced Inflammation by Reducing Toll-like Receptor 4 Expression in BV-2 Murine Microglial Cells
Source: Pharmaceuticals (Basel). 2025 Jul 24;18(8):1099. doi: 10.3390/ph18081099 (PMC12389435; doi:10.3390/ph18081099)
Supplement: Supplementary file 1 [file pharmaceuticals-18-01099-s001.zip › Table S2.pdf]

Table S2. Molecules belonging to each pathway category (ELN vs. control).

Ingenuity Canonical Pathways	-log(p-value)	Ratio	z-score	Molecules
Role of Hypercytokinemia/hyperchemokinemia in the Pathogenesis of Influenza	22.5	0.302	5.099	CCL2, CCL4, CCL5, CXCL10, CXCL3, EIF2AK2, IFIT2, IFIT3, IL1A, IL1RN, IL36G, IL6, IRF7, IRF9, ISG15, ISG20, NLRP3, OAS1, Oas1b, OAS2, OAS3, RIGI, RSAD2, STAT1, STAT2, TNF
Interferon Alpha/Beta Signaling	16.7	0.282	4.025	GBP2, HLA-A, IFI35, IFIT1, IFIT2, IFIT3, IRF7, IRF9, ISG15, ISG20, OAS1, OAS2, OAS3, OASL, RSAD2, SOCS1, STAT1, STAT2, USP18, XAF1
Activation of IRF by Cytosolic Pattern Recognition Receptors	15	0.277	2.183	CD40, DHX58, IFIH1, IFIT2, IKBKE, IL6, IRF7, IRF9, ISG15, LTA, NFKBIA, NFKBIE, RELB, RIGI, STAT1, STAT2, TNF, ZBP1
Pathogen Induced Cytokine Storm Signaling Pathway	14.2	0.0997	3.781	CCL2, CCL3L3, CCL4, CCL5, Ccl8, CCR5, CIITA, CLCF1, COL4A2, CXCL10, Cxcl3, CXCL3, CXCR4, DHX58, IFIH1, IL1A, IL1R1, IL1RL2, IL1RN, IL21R, IL36G, IL6, IRF7, IRF9, LTA, NLRC5, NLRP10, NLRP3, NOD2, Ppbp, RIGI, STAT1, STX11, TLR8, TNF, TNFSF10, ZBP1
Interferon gamma signaling	14.1	0.213	3.578	CIITA, GBP2, GBP4, GBP5, GBP6, GBP7, HLA-A, ICAM1, IRF7, IRF9, OAS1, OAS2, OAS3, OASL, SOCS1, STAT1, TRIM14, TRIM17, TRIM5, VCAM1
Interleukin-10 Signaling	13.9	0.333	2.84	CCL2, CCL4, CCL5, CCR5, CSF1, CSF3, CXCL10, FPR1, ICAM1, IL1A, IL1R1, IL1RN, IL6, PTGS2, TNF
Role of Pattern Recognition Receptors in Recognition of Bacteria and Viruses	11.6	0.141	3.162	CCL5, CLCF1, CLEC6A, EIF2AK2, IFIH1, IL1A, IL36G, IL6, IRF7, LTA, NLRP3, NOD2, OAS1, Oas1b, OAS2, OAS3, PTX3, RELB, RIGI, TLR8, TNF, TNFSF10
Macrophage Classical Activation Signaling Pathway	10.8	0.122	2.711	ACOD1, CCL5, CD40, CIITA, CLCF1, CXCL10, GBP2, GBP4, IL1A, IL36G, IL6, IRF9, LTA, MERTK, NFKBIA, NFKBIE, PARP14, PARP9, SOCS1, STAT1, STAT2, TNF, TNFSF10
TREM1 Signaling	9.12	0.182	1.387	CCL2, CD40, CD83, CIITA, CXCL3, ICAM1, IL6, NLRC5, NLRP10, NLRP3, NOD2, RELB, TLR8, TNF
Molecular Mechanisms of Cancer	9	0.0561	1.443	ADGRG1, ADORA2A, ADRA2A, Aph1c, C5AR2, CALCRL, CCR4, CCR5, CDKN2B, CHRM3, CNR2, CX3CR1, EDN1, EGF, FAS, FPR1, FPR2, GNAL, GPR141, GPR160, GPR162, GPR183, GPR84, HEY1, HGF, IL15RA, IL1A, IL1R1, IL1RL2, IL21R, ITGA3, ITGB3, MMP10, MMP12, MMP13, MMP2, NFKBIA, NFKBIE, PDGFB, PTGER1, RALGDS, RELB, RHOJ, RHOV, SHC3, SSTR5, VIPR1, VIPR2
Tumor Microenvironment Pathway	8.8	0.112	2.524	CCL2, CSF1, CSF3, CXCR4, EGF, FAS, FOXO6, HGF, HLA-A, ICAM1, IL6, ITGB3, MMP10, MMP12, MMP13, MMP2, PDGFB, PTGS2, RELB, TNF
Interferon Signaling	8.59	0.278	2.333	IFI35, IFIT1, IFIT3, IRF9, ISG15, OAS1, SOCS1, STAT1, STAT2, TAP1
Role of PKR in Interferon Induction and Antiviral Response	8.32	0.125	2.138	EIF2AK2, FAS, HSPA1A/HSPA1B, IFIH1, IKBKE, IRF9, NFKBIA, NFKBIE, NLRP10, NLRP3, PDGFB, PDGFRB, RELB, RIGI, STAT1, STAT2, TNF

Hematoma Resolution Signaling Pathway	8.1	0.0891	-1.877	C8G, CCR4, CLCF1, CX3CR1, FPR2, HMOX1, IL1A, IL36G, IL6, LTA, MERTK, MMP10, MMP12, MMP13, MMP2, NQO1, PTGER1, SOCS1, SOD2, STAT1, THBS1, TNF, TNFSF10
IL-33 Signaling Pathway	7.79	0.103	2.357	CASP4, CCL2, CCL5, H2BC17, ICAM1, IKBKE, IL1A, IL1RN, IL36G, IL6, LPIN1, MAPKAPK2, MMP12, MMP2, NFKBIA, NFKBIE, PTGS2, TNF, VCAM1
NOD1/2 Signaling Pathway	7.63	0.101	2.065	CCL2, CLCF1, HSPA1A/HSPA1B, IKBKE, IL1A, IL36G, IL6, IRF7, LTA, MAPKAPK2, NFKBIA, NFKBIE, NLRP3, NOD2, RIGI, TLR8, TNF, TNFAIP3, TNFSF10
Hepatic Fibrosis Signaling Pathway	7.59	0.0695	2.117	CACNG8, CCL2, CCL5, CD40, EDN1, GLIS1, ICAM1, IKBKE, IL1A, IL1R1, IL1RL2, IL1RN, IL36G, ITGA3, ITGB3, MMP13, MYL2, NFKBIA, NFKBIE, PDGFB, PDGFRB, RELB, RHOJ, RHOV, SERPINE1, SOD2, TNF, VCAM1, WNT10B
IL-10 Signaling	7.5	0.11	-1.213	ARG2, CCR5, HLA-A, HMOX1, ICAM1, IKBKE, IL1A, IL1R1, IL1RL2, IL1RN, IL36G, IL6, NFKBIA, NFKBIE, RELB, STAT1, TNF
CGAS-STING Signaling Pathway	7.3	0.114	2.5	ATP6V0D2, CCL2, CLCF1, ICAM1, IKBKE, IL1A, IL36G, IL6, LTA, NFKBIA, NLRP3, STAT1, TNF, TNFSF10, TREX1, ZBP1
Role of Chondrocytes in Rheumatoid Arthritis Signaling Pathway	7.26	0.113	3	CCL2, CXCR4, IL1A, IL1R1, IL1RL2, IL1RN, IL36G, IL6, LTA, MMP10, MMP12, MMP13, MMP2, NLRP3, PTGS2, TNF
Multiple Sclerosis Signaling Pathway	7.2	0.0901	2.683	C8G, CAPN3, CLCF1, FAS, HLA-A, IL1A, IL36G, IL6, LTA, MBP, NLRP3, PARP10, PARP12, PARP14, PARP9, RNF213, STAT1, TLR8, TNF, TNFSF10
ISGylation Signaling Pathway	7.16	0.13	2.673	DTX3L, EIF2AK2, IFIH1, IL6, IRF7, IRF9, ISG15, ITGA3, RIGI, SQSTM1, STAT1, STAT2, TLR8, USP18
Coronavirus Pathogenesis Pathway	7.1	0.0931	0.229	CCL2, CCL5, DDIT3, IL6, IRF7, IRF9, NFKBIA, NFKBIE, NLRP3, OAS1, Oas1b, OAS2, OAS3, PTGS2, RELB, RIGI, SERPINE1, STAT1, STAT2
Neuroinflammation Signaling Pathway	7.06	0.0757	1.964	Aph1c, CCL2, CCL5, CD40, CX3CR1, CXCL10, FAS, HLA-A, HMOX1, ICAM1, IKBKE, IL1R1, IL6, IRF7, KLK3, MR1, NLRP3, PTGS2, RELB, SOD2, STAT1, TLR8, TNF, VCAM1
Interleukin-4 and Interleukin-13 signaling	7.06	0.127	1.387	CCL2, FSCN1, HGF, HMOX1, ICAM1, IL1A, IL6, LCN2, MMP2, PTGS2, SOCS1, STAT1, TNF, VCAM1
Wound Healing Signaling Pathway	6.95	0.0833	2.837	CCL5, CLCF1, COL4A2, EGF, IL1A, IL1R1, IL1RL2, IL1RN, IL36G, IL6, ITGA3, LAMC2, LTA, MMP10, NFKBIA, NFKBIE, PDGFB, SHC3, STAT1, TNF, TNFSF10
CD40 Signaling	6.82	0.164	1	CD40, ICAM1, IKBKE, LTA, MAPKAPK2, NFKBIA, NFKBIE, PTGS2, RELB, TNFAIP3, TRAF1
TNFR2 Signaling	6.59	0.25	2	IKBKE, LTA, NFKBIA, NFKBIE, RELB, TNF, TNFAIP3, TRAF1
Differential Regulation of Cytokine Production in	6.47	0.304	2.646	CCL2, CCL4, CCL5, CSF3, IL1A, LCN2, TNF

Intestinal Epithelial Cells by IL-17A and IL-17F				
PPAR Signaling	6.36	0.121	-2.887	IKBKE, IL1A, IL1R1, IL1RL2, IL1RN, IL36G, NFKBIA, NFKBIE, PDGFB, PDGFRB, PTGS2, RELB, TNF
Acute Phase Response Signaling	6.33	0.0919	2.138	CFB, HMOX1, HP, IKBKE, IL1A, IL1R1, IL1RN, IL36G, IL6, NFKBIA, NFKBIE, RELB, Saa3, SERPINE1, SOCS1, SOD2, TNF
OAS Antiviral Response	6.31	0.556	2.236	OAS1, OAS2, OAS3, OASL, RIGI
Crosstalk between Dendritic Cells and Natural Killer Cells	6.31	0.132	2.121	CD28, CD40, CD83, FAS, FSCN1, HLA-A, IL15RA, IL6, LTA, RELB, TNF, TNFSF10
Pyroptosis Signaling Pathway	6.15	0.128	1.732	CASP4, GBP2, GBP4, GBP5, GBP7, IL1A, IL1R1, MEFV, NLRP10, NLRP3, TLR8, TNF
Role of Macrophages, Fibroblasts and Endothelial Cells in Rheumatoid Arthritis	6.09	0.0691	3.545	CCL2, CCL5, CSF1, ICAM1, IKBKE, IL1A, IL1R1, IL1RL2, IL1RN, IL36G, IL6, LTA, MAPKAPK2, MMP13, NFKBIA, NFKBIE, PDGFB, SOCS1, TLR8, TNF, TRAF1, VCAM1, WNT10B
Atherosclerosis Signaling	6.03	0.105	2.53	CCL2, CD40, CSF1, CXCR4, ICAM1, IL1A, IL1RN, IL36G, IL6, MMP13, PDGFB, RELB, TNF, VCAM1
Differential Regulation of Cytokine Production in Macrophages and T Helper Cells by IL-17A and IL-17F	5.88	0.333	2.449	CCL2, CCL4, CCL5, CSF3, IL6, TNF
NFE2L2 Regulating Anti-oxidant/Detoxification Enzymes	5.73	0.316	2.449	GCLM, HMOX1, NQO1, SLC7A11, SRXN1, TXNRD1
FXR/RXR Activation	5.59	0.0856	-2.5	CLCF1, DDIT3, GCLM, GSTO2, HMOX1, ICAM1, IL1A, IL1RN, IL36G, IL6, LTA, NLRP3, NQO1, PTGS2, TNF, TNFSF10
HMGB1 Signaling	5.54	0.0898	2.53	CCL2, CLCF1, ICAM1, IL1A, IL1R1, IL36G, IL6, LTA, RELB, RHOJ, RHOV, SERPINE1, TNF, TNFSF10, VCAM1
IL-6 Signaling	5.43	0.101	2.309	IKBKE, IL1A, IL1R1, IL1RL2, IL1RN, IL36G, IL6, MAPKAPK2, NFKBIA, NFKBIE, RELB, SOCS1, TNF
Cachexia Signaling Pathway	5.36	0.0625	2.711	CAPN3, CASP4, CCL2, CLCF1, EIF2AK2, FBXO32, FOXO6, GDF15, HSPA1A/HSPA1B, IKBKE, IL1A, IL1R1, IL1RL2, IL1RN, IL36G, IL6, LCN2, LTA, STAT1, STAT2, TLR8, TNF, TNFSF10
Role of RIG1-like Receptors in Antiviral Innate Immunity	5.31	0.174	1.342	DHX58, IFIH1, IKBKE, IRF7, NFKBIA, NFKBIE, RELB, RIGI
Toll-like Receptor Signaling	5.24	0.128	1.633	EIF2AK2, IL1A, IL1RN, IL36G, NFKBIA, RELB, TLR8, TNF, TNFAIP3, TRAF1
Death Receptor Signaling	5.22	0.115	1.897	FAS, IKBKE, NFKBIA, NFKBIE, PARP10, PARP12, PARP14, PARP9, RELB, TNF, TNFSF10
IL-17A Signaling in Fibroblasts	5.14	0.125	2.53	CCL2, IKBKE, IL6, LCN2, NFKBIA, NFKBIE, RELB, SERPINH1, TNF, VCAM1
Airway Pathology in Chronic Obstructive Pulmonary Disease	5.1	0.102	1	C8G, CCL2, CLCF1, CXCL3, IL1A, IL36G, IL6, LCN2, LTA, MMP2, TNF, TNFSF10

Th1 Pathway	4.95	0.0984	0	Aph1c, CCR5, CD28, CD40, HLA-A, ICAM1, ICOS, IL27, IL6, LTA, SOCS1, STAT1
Activin Inhibin Signaling Pathway	4.92	0.0758	1.5	CXCR4, FBXO32, FOSL1, IKBKE, IL1A, IL1R1, IL1RL2, IL1RN, IL36G, IL6, MMP2, NFKBIA, PTGS2, SERPINE1, TLR8, TNF
Macrophage Alternative Activation Signaling Pathway	4.85	0.0789	0.775	ADORA2A, CIITA, CSF1, FPR1, IL1A, IL1RN, IL36G, IL6, NFKBIA, NFKBIE, RELB, SOCS1, STAT1, THBS1, TNF
Class A/1 (Rhodopsin-like receptors)	4.79	0.0631	1.342	ADORA2A, ADRA2A, C5AR2, CCL2, CCL4, CCL5, CCR4, CCR5, CHRM3, CNR2, CX3CR1, CXCL10, CXCL3, CXCR4, EDN1, FPR1, FPR2, GPR183, PTGER1, SSTR5
IL-27 Signaling Pathway	4.74	0.0938	0	CD28, CD40, HLA-A, IL1R1, IL1RL2, IL27, MR1, NLRP3, PTGS2, RELB, STAT1, TLR8
S100 Family Signaling Pathway	4.64	0.0453	1.521	ADGRG1, ADORA2A, ADRA2A, C5AR2, CACNG8, CALCRL, CCL4, CCR4, CCR5, CHRM3, CNR2, CX3CR1, EGF, EZR, FPR1, FPR2, GPR141, GPR160, GPR162, GPR183, GPR84, IL6, KLK3, MMP10, MMP12, MMP13, MMP2, PTGER1, SSTR5, TNF, VCAM1, VDR, VIPR1, VIPR2, WNT10B
Pathogenesis of Multiple Sclerosis	4.64	0.444	1	CCL4, CCL5, CCR5, CXCL10
PI3K/AKT Signaling	4.59	0.075	1.89	EPM2A, GDF15, IKBKE, IL15RA, IL1R1, IL1RL2, IL21R, INPP5J, ITGA3, ITGB3, NFKBIA, NFKBIE, PTGS2, RELB, SFN
Role of Osteoclasts in Rheumatoid Arthritis Signaling Pathway	4.45	0.0617	2.828	COL4A2, CSF1, FOXO6, IKBKE, IL1R1, IL1RL2, ITGB3, LCP2, MITF, MMP10, MMP12, MMP13, MMP2, NFKBIA, NFKBIE, RHOJ, RHOV, SHC3, TNF
HIF1α Signaling	4.37	0.0718	1.807	CCNG2, EDN1, EGF, HGF, HMOX1, HSPA1A/HSPA1B, IL6, MET, MKNK2, MMP10, MMP12, MMP13, MMP2, PDGFB, SERPINE1
Role of MAPK Signaling in Inhibiting the Pathogenesis of Influenza	4.36	0.114	1.667	CCL2, CCL5, CXCL10, EIF2AK2, IL6, NFKBIA, NFKBIE, PTGS2, TNF
CREB Signaling in Neurons	4.34	0.0478	0.962	ADGRG1, ADORA2A, ADRA2A, C5AR2, CACNG8, CALCRL, CCR4, CCR5, CHRM3, CNR2, CX3CR1, EGF, FPR1, FPR2, GNAL, GPR141, GPR160, GPR162, GPR183, GPR84, HGF, NTRK1, PDGFB, PDGFRB, PTGER1, SHC3, SSTR5, VIPR1, VIPR2
IL-17 Signaling	4.32	0.0749	3.742	CCL2, CLCF1, CSF3, CXCL3, IL1A, IL36G, IL6, LCN2, LTA, MMP13, MMP2, PTGS2, TNF, TNFSF10
p38 MAPK Signaling	4.3	0.0917	2.111	DDIT3, FAS, IL1A, IL1R1, IL1RL2, IL1RN, IL36G, MAPKAPK2, MKNK2, STAT1, TNF
LXR/RXR Activation	4.2	0.0894	-1.897	ARG2, CCL2, IL1A, IL1R1, IL1RL2, IL1RN, IL36G, IL6, PTGS2, RELB, TNF
IL-15 Production	4.2	0.0894	1.265	FGR, IL6, LMTK3, MATK, MERTK, MET, NTRK1, PDGFRB, RELB, STAT1, TXK
Induction of Apoptosis by HIV1	4.17	0.123	0.378	CXCR4, FAS, IKBKE, NFKBIA, NFKBIE, RELB, TNF, TRAF1

Colorectal Cancer Metastasis Signaling	4.13	0.0627	2.84	EGF, GNAL, IL6, MMP10, MMP12, MMP13, MMP2, PTGER1, PTGS2, RALGDS, RELB, RHOJ, RHOV, STAT1, TLR8, TNF, WNT10B
FAT10 Cancer Signaling Pathway	4.08	0.14	1.342	CXCR4, IKBKE, IL6, NFKBIA, NFKBIE, RELB, TNF
NAFLD Signaling Pathway	3.99	0.0664	2.84	CLCF1, DDIT3, EPM2A, IKBKE, IL1A, IL36G, IL6, LPIN1, LTA, NLRP3, PTGS2, SOCS1, TLR8, TNF, TNFSF10
G-Protein Coupled Receptor Signaling	3.96	0.0441	0.898	ADGRG1, ADORA2A, ADRA2A, C5AR2, CALCRL, CCR4, CCR5, CHRM3, CNR2, CX3CR1, FOXO6, FPR1, FPR2, GNAL, GPR141, GPR160, GPR162, GPR183, GPR84, IKBKE, MYL2, NFKBIA, NFKBIE, PDE8B, PTGER1, RELB, RGS18, SHC3, SSTR5, VIPR1, VIPR2
STAT3 Pathway	3.84	0.0815	1.134	EGF, HGF, IL15RA, IL1A, IL1R1, IL1RL2, IL21R, NTRK1, PDGFB, PDGFRB, SOCS1
Cellular Effects of Sildenafil (Viagra)	3.76	0.0436	-0.73	ADGRG1, ADORA2A, ADRA2A, ATP2A3, C5AR2, CACNG8, CALCRL, CAPN3, CCR4, CCR5, CHRM3, CNR2, CX3CR1, FAS, FPR1, FPR2, GPR141, GPR160, GPR162, GPR183, GPR84, IL6, MYL2, NFKBIA, PTGER1, SOD2, SSTR5, TNF, VIPR1, VIPR2
Role of Osteoblasts in Rheumatoid Arthritis Signaling Pathway	3.62	0.0615	3.357	CLCF1, IL1A, IL36G, IL6, LTA, MMP10, MMP12, MMP13, MMP2, PTGS2, STAT1, STAT2, TNF, TNFSF10, WNT10B
Regulation of the Epithelial Mesenchymal Transition by Growth Factors Pathway	3.62	0.0677	2.714	EGF, HGF, IKBKE, IL6, LTA, MET, MMP2, PDGFB, PDGFRB, RELB, SHC3, TNF, TNFSF10
NF-κB Activation by Viruses	3.61	0.103	1.633	CCR5, EIF2AK2, IKBKE, ITGA3, ITGB3, NFKBIA, NFKBIE, RELB
Cardiac Hypertrophy Signaling (Enhanced)	3.59	0.0461	2.524	ADRA2A, ATP2A3, CACNG8, CLCF1, EDN1, IKBKE, IL15RA, IL1A, IL1R1, IL1RL2, IL21R, IL36G, IL6, ITGA3, ITGB3, LTA, MAPKAPK2, MKNK2, PDE8B, PTGS2, RELB, TG, TNF, TNFSF10, WNT10B
DDX58/IFIH1-mediated Induction of Interferon-Alpha/Beta	3.57	0.101	2.828	IFIH1, IKBKE, IRF7, ISG15, NFKBIA, NLRC5, RIGI, TNFAIP3
Hepatic Cholestasis	3.51	0.0628	3.207	CLCF1, IKBKE, IL1A, IL1R1, IL1RL2, IL1RN, IL36G, IL6, LTA, NFKBIA, NFKBIE, RELB, TNF, TNFSF10
BBSome Signaling Pathway	3.46	0.047	0.209	ADGRG1, ADORA2A, ADRA2A, C5AR2, CALCRL, CCR4, CCR5, CHRM3, CNR2, CX3CR1, FPR1, FPR2, GLIS1, GPR141, GPR160, GPR162, GPR183, GPR84, IFT70A, PTGER1, SSTR5, VIPR1, VIPR2
Interleukin-1 Family Signaling	3.37	0.0775	2.53	IL18BP, IL1A, IL1R1, IL1RL2, IL1RN, IL36G, NFKBIA, NOD2, PTPN14, SQSTM1
G alpha (i) Signaling Events	3.36	0.0581	0.258	ADRA2A, CCL4, CCL5, CCR4, CCR5, CNR2, CX3CR1, CXCL10, CXCL3, CXCR4, FPR1, FPR2, GPR183, RGS18, SSTR5
Breast Cancer Regulation by Stathmin1	3.36	0.0438	0.6	ADGRG1, ADORA2A, ADRA2A, C5AR2, CALCRL, CCR4, CCR5, CHRM3, CNR2, CX3CR1, EGF, FPR1, FPR2, GPR141, GPR160,

				GPR162, GPR183, GPR84, HGF, MMP2, PDGFB, PTGER1, SHC3, SSTR5, VIPR1, VIPR2
Role of IL-17F in Allergic Inflammatory Airway Diseases	3.35	0.128	2	CCL2, CCL4, CXCL10, IL6, MMP13, RELB
Necroptosis Signaling Pathway	3.32	0.071	2.111	CAPN3, EIF2AK2, FAS, IRF9, MERTK, NLRP3, STAT1, STAT2, TNF, TNFSF10, ZBP1
Ferroptosis Signaling Pathway	3.31	0.0763	-1.265	ANGPTL4, CHAC1, GCH1, H2BC17, HMOX1, SLC38A1, SLC3A2, SLC7A11, SQSTM1, TXNRD1
IL-8 Signaling	3.24	0.0619	1.265	GNAL, HMOX1, ICAM1, IKBKE, ITGB3, MMP2, MYL2, NFKBIA, NFKBIE, PTGS2, RHOJ, RHOV, VCAM1
TNFR1 Signaling	3.15	0.118	2	IKBKE, NFKBIA, NFKBIE, RELB, TNF, TNFAIP3
Apelin Liver Signaling Pathway	3.13	0.2	1	EDN1, FAS, PDGFRB, TNF
PIP3 Activates AKT Signaling	3.09	0.0714	0.632	CD28, EGF, FOXO6, HGF, ICOS, MET, NRG4, PDGFB, PDGFRB, TRIB3
Phagosome Formation	3.01	0.0402	0.378	ADGRG1, ADORA2A, ADRA2A, C5AR2, CALCRL, CCR4, CCR5, CHRM3, CLEC4E, CNR2, CX3CR1, FGR, FPR1, FPR2, GPR141, GPR160, GPR162, GPR183, GPR84, HMOX1, ITGA3, ITGB3, MYL2, PTGER1, SSTR5, TLR8, VIPR1, VIPR2
Gαq Signaling	2.98	0.0647	1.633	CHRM3, EPM2A, GNAL, HMOX1, IKBKE, NFKBIA, NFKBIE, RELB, RGS18, RHOJ, RHOV
TWEAK Signaling	2.98	0.135	-1	IKBKE, NFKBIA, NFKBIE, RELB, TRAF1
Lymphotoxin β Receptor Signaling	2.98	0.109	1.342	IKBKE, LTA, NFKBIA, RELB, TRAF1, VCAM1
Serotonin Receptor Signaling	2.94	0.0448	1.091	ADRA2A, CACNG8, CD38, EDN1, FGR, GCH1, GNAL, HMOX1, IL6, ITGB3, KALRN, MMP2, MYL2, NFKBIA, PTGS2, RHOJ, RHOV, SLC6A4, SQSTM1, TGM2, TNF
NLR signaling pathways	2.94	0.107	2.449	CASP4, HMOX1, MEFV, NLRP3, NOD2, TNFAIP3
IL-12 Signaling and Production in Macrophages	2.91	0.057	2.496	ADRA2A, CCR5, CD40, DHX58, IFIH1, IKBKE, NOD2, RELB, RIGI, STAT1, THBS1, TNF, VDR
TNF Signaling	2.89	0.105	1.633	IKBKE, MAPKAPK2, SMPD3, TNF, TNFAIP3, TRAF1
Inhibition of Matrix Metalloproteases	2.88	0.128	-2.236	MMP10, MMP12, MMP13, MMP2, TIMP2
Antigen Presentation Pathway	2.88	0.128	-0.447	CIITA, HLA-A, MR1, NLRC5, TAP1
Erythropoietin Signaling Pathway	2.86	0.0625	-3	CLCF1, IL1A, IL36G, IL6, LTA, NFKBIA, NFKBIE, RELB, SHC3, TNF, TNFSF10
NAD Signaling Pathway	2.84	0.0662	1.897	CD38, EGF, H2BC17, HGF, PARP10, PARP12, PARP14, PARP9, PDGFB, SOD2
Role of Tissue Factor in Cancer	2.79	0.058	2.309	CSF1, EGF, FGR, HGF, IKBKE, ITGA3, ITGB3, MET, MMP13, MMP2, PTGS2, TNF
cAMP-mediated Signaling	2.78	0.0551	1.155	ADORA2A, ADRA2A, AKAP3, CCR4, CHRM3, CNR2, FPR1, FPR2, GNAL, PDE8B, RGS18, VIPR1, VIPR2

Osteoarthritis Pathway	2.78	0.0551	2.53	CASP4, GLIS1, IL1R1, IL1RL2, ITGA3, ITGB3, MMP10, MMP12, MMP13, PTGS2, RELB, SDC4, TNF
Transport of Inorganic Cations/Anions and Amino Acids/Oligopeptides	2.78	0.0769	-0.707	SLC26A11, SLC26A4, SLC32A1, SLC38A1, SLC3A1, SLC3A2, SLC7A11, SLC9A7
Apoptosis Signaling	2.78	0.0769	0.378	BCL2A1, CAPN3, FAS, IKBKE, NFKBIA, NFKBIE, RELB, TNF
Systemic Lupus Erythematosus in B Cell Signaling Pathway	2.76	0.0387	3.53	CD40, CD79B, CLCF1, FGR, FOXO6, IFIH1, IFIT2, IFIT3, Ighv1-58, IL1A, IL36G, IL6, INPP5J, IRF7, IRF9, ISG15, ISG20, LILRB4, LTA, RASGRP3, RELB, SHC3, STAT1, STAT2, TLR8, TNF, TNFSF10, TRAF1
April Mediated Signaling	2.73	0.119	0	IKBKE, NFKBIA, NFKBIE, RELB, TRAF1
Extracellular Matrix Organization	2.72	0.0755	1.414	CEACAM1, COL4A2, ITGA3, ITGB3, LAMC2, PDGFB, SERPINE1, TNN
Xenobiotic Metabolism AHR Signaling Pathway	2.6	0.0805	1.633	GSTO2, IL1A, IL6, NQO1, RELB, TNF, UGT1A9 (includes others)
Sleep REM Signaling Pathway	2.6	0.0721	-2.121	CHRM3, CLCF1, IL1A, IL36G, IL6, LTA, TNF, TNFSF10
Autism Signaling Pathway	2.59	0.0487	2.324	CACNG8, CCL2, CLCF1, HGF, HLA-A, IL1A, IL1RN, IL36G, IL6, LTA, MET, MR1, TNF, TNFSF10, WNT10B
HOTAIR Regulatory Pathway	2.59	0.0613	1.667	ICAM1, MET, MEX3B, MMP10, MMP12, MMP13, MMP2, NFKBIA, RELB, WNT10B
Th2 Pathway	2.58	0.0657	-1.134	Aph1c, CCR4, CCR5, CD28, CD40, CXCR4, HLA-A, ICAM1, ICOS
Cytosolic Sensors of Pathogen-Associated DNA	2.55	0.109	2.236	IFI16, IRF7, NFKBIA, TREX1, ZBP1
Leukocyte Extravasation Signaling	2.55	0.057	2.714	CXCR4, EZR, ICAM1, ITGA3, MMP10, MMP12, MMP13, MMP2, TIMP2, TXK, VCAM1
IL-17A Signaling in Airway Cells	2.53	0.0896	0.447	CXCL3, IKBKE, IL6, NFKBIA, NFKBIE, RELB
Antioxidant Action of Vitamin C	2.53	0.0702	-2.236	GSTO2, HMOX1, IKBKE, NFKBIA, NFKBIE, RELB, TNF, TXNRD1
Immunogenic Cell Death Signaling Pathway	2.52	0.0778	2.646	CXCL10, DDIT3, FPR1, HSPA1A/HSPA1B, IL6, NLRP3, TNF
iNOS Signaling	2.51	0.106	2	IKBKE, NFKBIA, NFKBIE, RELB, STAT1
FOXO-mediated Transcription of Oxidative Stress, Metabolic and Neuronal Genes	2.45	0.133	-1	ABCA6, FBXO32, FOXO6, SOD2
Pulmonary Healing Signaling Pathway	2.45	0.0553	2.111	CXCR4, EGF, FGR, MMP10, MMP12, MMP13, MMP2, NFKBIA, THBS1, TNF, WNT10B
Adrenergic Receptor Signaling Pathway (Enhanced)	2.43	0.055	-2.714	ADRA2A, ATP6V0D2, CACNG8, CLCF1, GNAL, IL1A, IL36G, IL6, LTA, TNF, TNFSF10
Regulation of Lipid Metabolism by PPARalpha	2.41	0.0672	1.414	ACSL1, ANGPTL4, HELZ2, ME1, NR1D1, TRIB3, TXNRD1, UGT1A9 (includes others)
MSP-RON Signaling in Macrophages Pathway	2.41	0.0672	-1.134	CIITA, IKBKE, KLK3, PTGS2, RELB, SOCS1, STAT1, TNF

Signaling by CSF1 (M-CSF) in myeloid cells	2.4	0.129	0	CSF1, GAB3, GRAP2, STAT1
CDX Gastrointestinal Cancer Signaling Pathway	2.4	0.0545	-2.53	CLCF1, IKBKE, IL1A, IL36G, IL6, LTA, PTGS2, RELB, TNF, TNFSF10, WNT10B
RAF/MAP Kinase Cascade	2.39	0.0496	0.832	DUSP8, EGF, HGF, ITGB3, KSR2, MET, NRG4, PDGFB, PDGFRB, RALGDS, RASA3, RASGRP3, SHC3
Small Cell Lung Cancer Signaling	2.36	0.0729	1	CDKN2B, IKBKE, NFKBIA, NFKBIE, PTGS2, RELB, TRAF1
IL-1 Signaling	2.36	0.0729	1.342	GNAL, IKBKE, IL1A, IL1R1, NFKBIA, NFKBIE, RELB
RAR Activation	2.31	0.042	-1.414	CDKN2B, CLCF1, DHRS3, EGF, IL1A, IL36G, IL6, LTA, MMP13, MMP2, PDE8B, RELB, RHOJ, RHOV, TGM2, TNF, TNFSF10, ZBTB16
Activation of Matrix Metalloproteinases	2.3	0.121	1	MMP10, MMP13, MMP2, TIMP2
Type II Diabetes Mellitus Signaling	2.27	0.0588	2	ACSL1, CACNG8, IKBKE, NFKBIA, NFKBIE, RELB, SMPD3, SOCS1, TNF
Cell Surface Interactions at the Vascular Wall	2.26	0.0521	0.302	CD2, CEACAM1, ITGA3, ITGB3, MERTK, OLR1, PROCR, SDC4, SLC3A2, SLC7A11, THBD
Parkinson's Signaling Pathway	2.21	0.0456	3.207	ATP6V0D2, CACNG8, CLCF1, CNR2, DDIT3, IL1A, IL36G, IL6, LTA, NLRP3, NQO1, TLR8, TNF, TNFSF10
VDR/RXR Activation	2.21	0.0769	1	CAMP, CCL5, CXCL10, HSD17B2, THBD, VDR
Docosahexaenoic Acid (DHA) Signaling	2.13	0.048	1.155	Aph1c, BCL2A1, EGF, FAS, HGF, HMOX1, OSGIN1, PDGFB, PTGS2, SYT16, SYT7, TNF
Chemokine Signaling	2.13	0.0741	0	CCL2, CCL4, CCL5, CCR5, CXCR4, MYL2
Signaling by PDGF	2.12	0.0862	1.342	COL4A2, PDGFB, PDGFRB, STAT1, THBS1
FAK Signaling	2.11	0.0329	1.372	ADGRG1, ADORA2A, ADRA2A, C5AR2, CALCRL, CAPN3, CCR4, CCR5, CHRM3, CNR2, CX3CR1, EGF, FPR1, FPR2, GPR141, GPR160, GPR162, GPR183, GPR84, IL15RA, IL1R1, IL1RL2, IL21R, ITGA3, ITGB3, MET, MMP2, PDGFRB, PTGER1, SH2D2A, SOCS1, SSTR5, VIPR1, VIPR2
Retinoic acid Mediated Apoptosis Signaling	2.06	0.0833	2.236	PARP10, PARP12, PARP14, PARP9, TNFSF10
Integrin Cell Surface Interactions	2.03	0.0706	0.816	COL4A2, ICAM1, ITGA3, ITGB3, THBS1, VCAM1
Collagen degradation	2.03	0.082	2.236	COL4A2, MMP10, MMP12, MMP13, MMP2
Gαi Signaling	2	0.0571	0.447	ADRA2A, CCR4, CNR2, FPR1, FPR2, GNAL, RALGDS, SHC3
Pulmonary Fibrosis Idiopathic Signaling Pathway	1.99	0.0429	3.051	COL4A2, EDN1, FOXO6, IL6, MMP10, MMP12, MMP13, MMP2, PDGFB, PDGFRB, RELB, SERPINE1, THBS1, WNT10B
Adrenomedullin signaling pathway	1.99	0.0503	1.667	CALCRL, IL1A, IL1RN, IL36G, LTA, MATK, MMP2, RELB, SHC3, TNF
Ribonucleotide Reductase Signaling Pathway	1.96	0.0526	2.333	HGF, IL6, MET, MMP2, PARP10, PARP12, PARP14, PARP9, THBS1

NRF2-mediated Oxidative Stress Response	1.9	0.0464	1.89	AOX1, CYP2S1, FOSL1, GCLM, GSTO2, HMOX1, MAFF, NQO1, SOD2, SQSTM1, TXNRD1
Degradation of the extracellular matrix	1.89	0.0758	2.236	LAMC2, MMP10, MMP12, MMP13, MMP2
Role of PI3K/AKT Signaling in the Pathogenesis of Influenza	1.89	0.0758	-1	CCL5, CCR5, NFKBIA, NFKBIE, RELB
Endocannabinoid Cancer Inhibition Pathway	1.88	0.0544	0	CASP4, CNR2, DDIT3, GNAL, MMP2, NUPR1, SMPD3, TRIB3
Cholecystokinin/Gastrin-mediated Signaling	1.87	0.0588	1.89	IL1A, IL1RN, IL36G, PTGS2, RHOJ, RHOV, TNF
TAK1-dependent IKK and NF-kappa-B Activation	1.86	0.0909	2	NFKBIA, NLRC5, NOD2, USP18
PTEN Signaling	1.81	0.053	-1.633	FOXO6, IKBKE, INPP5J, ITGA3, ITGB3, NTRK1, PDGFRB, RELB
ISG15 Antiviral Mechanism	1.76	0.0704	2.236	EIF2AK2, IFIT1, ISG15, RIGI, STAT1
Pancreatic Adenocarcinoma Signaling	1.75	0.0556	2.236	CDKN2B, EGF, HMOX1, PTGS2, RALGDS, RELB, STAT1
Glioma Invasiveness Signaling	1.71	0.0685	0.447	ITGB3, MMP2, RHOJ, RHOV, TIMP2
Transcriptional Activity of SMAD2/SMAD3:SMAD4 Heterotrimer	1.7	0.0816	1	CDKN2B, SERPINE1, STAT1, TFDP2
Aryl Hydrocarbon Receptor Signaling	1.69	0.0503	0.447	FAS, GSTO2, IL1A, IL6, NQO1, RELB, TGM2, TNF
ERK5 Signaling	1.69	0.0676	2	EGF, FOSL1, NTRK1, SFN, SH2D2A
Production of Nitric Oxide and Reactive Oxygen Species in Macrophages	1.68	0.0471	1.414	ARG2, IKBKE, NFKBIA, NFKBIE, RELB, RHOJ, RHOV, STAT1, TNF
Signaling by NOTCH3	1.67	0.08	0	Aph1c, EGF, HEY1, STAT1
Myelination Signaling Pathway	1.64	0.0398	0.832	Aph1c, CYTH1, FGR, GLIS1, ITGA3, ITGB3, KLK3, LAMC2, MBP, PDGFB, PDGFRB, WNT10B, XAF1
HER-2 Signaling in Breast Cancer	1.63	0.0441	1.265	EGF, FGR, IKBKE, IL1A, ITGB3, MMP2, NFKBIA, NFKBIE, PTGS2, RELB
IL-7 Signaling Pathway	1.6	0.0641	0	FOXO6, HGF, MET, SOCS1, STAT1
Neutrophil Degranulation	1.58	0.0357	2.668	AMPD3, CAMP, CEACAM1, FGR, FPR1, FPR2, GPR84, HP, HSPA1A/HSPA1B, LCN2, OLR1, PTX3, RAB44, SERPINB12, SLPI, TARM1, TIMP2
Role of JAK Family Kinases in IL-6-type Cytokine Signaling	1.58	0.0633	1.342	CLCF1, IL27, IL6, SOCS1, STAT1
Senescence Pathway	1.58	0.0401	0.905	CACNG8, CAPN3, CDKN2B, IKBKE, IL1A, IL6, MAPKAPK2, Saa3, SERPINE1, SOD2, SQSTM1, YPEL3
Signaling by NTRK1 (TRKA)	1.54	0.0617	2.236	ADORA2A, MAPKAPK2, NTRK1, RALGDS, SHC3
JAK/STAT Signaling	1.52	0.061	1	IL6, RELB, SOCS1, STAT1, STAT2

Oxidative Stress Induced Senescence	1.44	0.0581	0	CDKN2B, H2BC17, MAPKAPK2, mir-24 (includes others), TFDP2
PDGF Signaling	1.43	0.0575	1.342	EIF2AK2, INPP5J, PDGFB, PDGFRB, STAT1
PAK Signaling	1.42	0.0513	1.342	ITGA3, ITGB3, MYL2, PDGFB, PDGFRB, TNF
CLEAR Signaling Pathway	1.38	0.0386	-0.905	ATP6V0D2, EGF, HGF, MITF, NTRK1, PDGFB, PDGFRB, SFN, SGSH, TLR8, TNF
Sphingosine-1-phosphate Signaling	1.37	0.05	0	CASP4, PDGFB, PDGFRB, RHOJ, RHOV, SMPD3
Renin-Angiotensin Signaling	1.36	0.0496	2	CCL2, CCL5, RELB, SHC3, STAT1, TNF
Dendritic Cell Maturation	1.29	0.0319	2.524	CD40, CD83, FSCN1, HLA-A, ICAM1, IKBKE, IL1A, IL1RL2, IL1RN, IL36G, IL6, LTA, MR1, NFKBIA, NFKBIE, RELB, STAT1, STAT2, TNF
Estrogen Receptor Signaling	1.26	0.0342	2.309	ARG2, CACNG8, EGF, FBXO32, FOXO6, GNAL, MMP10, MMP12, MMP13, MMP2, MYL2, RELB, SHC3, SOD2
Acetylcholine Receptor Signaling Pathway	1.26	0.0412	0	Aph1c, CACNG8, CASP4, CHRM3, GNAL, HMOX1, IL6, TNF
PPAR α/RXR α Activation	1.26	0.0412	-1	HELZ2, IKBKE, IL1R1, IL1RL2, IL6, NFKBIA, NFKBIE, RELB
HEY1 Signaling Pathway	1.25	0.0435	0.378	Aph1c, HEY1, IL6, MMP10, MMP12, MMP13, MMP2
Role of JAK1 and JAK3 in γc Cytokine Signaling	1.23	0.058	1	IL15RA, IL21R, SOCS1, STAT1
p75 NTR Receptor-Mediated Signaling	1.21	0.05	0.447	Aph1c, ARHGEF40, KALRN, NFKBIA, SQSTM1
Oxytocin in Brain Signaling Pathway	1.21	0.0402	-0.707	CACNG8, DDIT3, GNAL, IL6, NLRP10, NLRP3, PTGS2, TNF
Response to elevated platelet cytosolic Ca2+	1.21	0.0455	0	EGF, HGF, ITGB3, PDGFB, SERPINE1, THBS1
HGF Signaling	1.21	0.0455	1	HGF, IL6, ITGA3, ITGB3, MET, PTGS2
Sertoli Cell-Germ Cell Junction Signaling Pathway (Enhanced)	1.19	0.0381	1	FOSL1, ICAM1, IL1A, IL1R1, IL1RL2, ITGA3, LAMC2, MMP2, TNF
Senescence-Associated Secretory Phenotype (SASP)	1.16	0.0548	1	CDKN2B, H2BC17, IL1A, IL6
Glioblastoma Multiforme Signaling	1.14	0.0409	1.633	EGF, PDGFB, PDGFRB, RHOJ, RHOV, SHC3, WNT10B
Ephrin A Signaling	1.12	0.0432	-0.816	Aph1c, EGF, FGR, HGF, MYL2, PDGFB
PD-1, PD-L1 cancer immunotherapy pathway	1.11	0.0467	1.342	CD28, HLA-A, LCP2, MR1, TNF
Costimulation by the CD28 Family	1.08	0.0513	-1	CD28, GRAP2, ICOS, TRIB3
Neutrophil Extracellular Trap Signaling Pathway	1.07	0.0325	1.941	ARG2, CAMP, CASP4, CCL5, CCR5, COL4A2, FGR, FPR1, ITGA3, MMP2, NLRP3, TLR8, TNF
Signaling by MET	1.06	0.0506	1	HGF, ITGA3, LAMC2, MET
G alpha (s) Signaling Events	1.05	0.0414	1.633	ADORA2A, CALCRL, GPR84, PDE8B, VIPR1, VIPR2
Glycosaminoglycan metabolism	1.05	0.05	-1	CSPG5, SDC4, SGSH, STAB2

Autophagy	1.04	0.0369	0.707	DDIT3, EGF, HGF, NOD2, PDGFB, SLC3A2, SQSTM1, TNF
RHOGDI Signaling	1.01	0.0364	-0.447	EZR, GNAL, GRIP1, ITGA3, ITGB3, MYL2, RHOJ, RHOV
WNT/SHH Axonal Guidance Signaling Pathway	1.01	0.0403	2.449	FGR, GLIS1, SEMA3C, SEMA3G, SFN, WNT10B
Regulation of Actin-based Motility by Rho	1.01	0.0435	0	ITGA3, ITGB3, MYL2, RHOJ, RHOV
Transcriptional Regulation of White Adipocyte Differentiation	0.988	0.0476	-1	ANGPTL4, HELZ2, TNF, WNT10B
Deubiquitination	0.966	0.0342	2.333	H2BC17, IFIH1, NFKBIA, NLRP3, NOD2, RIGI, TNFAIP3, TNIP3, USP18
Signaling by Rho Family GTPases	0.937	0.0337	0.816	CDC42EP2, EZR, GNAL, ITGA3, ITGB3, MYL2, RELB, RHOJ, RHOV
Regulation of Insulin-like Growth Factor (IGF) Transport and Uptake by IGFBPs	0.908	0.0403	1.342	CSF1, IL6, KLK3, MMP2, VWA1
Gαs Signaling	0.887	0.0397	1	ADORA2A, CHRM3, GNAL, VIPR1, VIPR2
MAPK6/MAPK4 Signaling	0.882	0.0435	2	CDC42EP2, KALRN, MMP10, MMP2
GP6 Signaling Pathway	0.876	0.0394	1	COL4A2, GRAP2, ITGB3, LAMC2, LCP2
ILK Signaling	0.866	0.0348	0	ITGB3, MYL2, PTGS2, RELB, RHOJ, RHOV, TNF
Immunoregulatory Interactions Between a Lymphoid and a Non-Lymphoid Cell	0.859	0.0347	1.134	CD40, HLA-A, ICAM1, ITGA3, LILRB4, PIANP, VCAM1
Ephrin Receptor Signaling	0.859	0.0347	0	CXCR4, EGF, GNAL, ITGA3, ITGB3, KALRN, PDGFB
Fcγ Receptor-mediated Phagocytosis in Macrophages and Monocytes	0.857	0.0426	2	EZR, FGR, HMOX1, LCP2
Class B/2 (Secretin family receptors)	0.834	0.0417	1	CALCRL, VIPR1, VIPR2, WNT10B
NF-κB Signaling	0.803	0.028	1.5	CD40, EGF, EIF2AK2, IL1A, IL1R1, IL1RN, IL36G, LTA, NFKBIA, NFKBIE, NTRK1, PDGFRB, RELB, TLR8, TNF, TNFAIP3
Pancreatic Secretion Signaling Pathway	0.802	0.0323	1.414	ADORA2A, ATP2A3, CD38, CHRM3, EGF, RASGRP3, VIPR1, VIPR2
Integrin Signaling	0.783	0.033	-0.378	CAPN3, ITGA3, ITGB3, MYL2, PDGFB, RHOJ, RHOV
G alpha (q) Signaling Events	0.782	0.0345	1.633	CHRM3, EDN1, FPR2, KALRN, PTGER1, RGS18
Protein Kinase A Signaling	0.782	0.0292	0.816	AKAP3, DUSP8, EPM2A, MTMR7, MYL2, NFKBIA, NFKBIE, PDE8B, PTGS2, PTPN14, RELB, SFN
Adipogenesis pathway	0.762	0.036	-1.342	DDIT3, LPIN1, NR1D1, TNF, WNT10B
MSP-RON Signaling in Cancer Cells Pathway	0.753	0.0357	0	KLK3, MET, NFKBIA, RELB, SFN
Apelin Endothelial Signaling Pathway	0.745	0.0355	1	CCL2, GNAL, ICAM1, RELB, VCAM1
Cardiac Hypertrophy Signaling	0.719	0.0307	0	ADRA2A, CACNG8, GNAL, IL6, MAPKAPK2, MYL2, RHOJ, RHOV

Ion channel transport	0.714	0.0328	0	ATP13A4, ATP2A3, ATP6V0D2, CLCN2, MCOLN2, TRPM3
Signaling by VEGF	0.707	0.037	1	ITGB3, MAPKAPK2, SH2D2A, TRIB3
Endocannabinoid Neuronal Synapse Pathway	0.679	0.0336	0	CACNG8, CNR2, GNAL, MGLL, PTGS2
Prostate Cancer Signaling	0.652	0.0351	1	KLK3, NFKBIA, NFKBIE, RELB
Role of MAPK Signaling in Promoting the Pathogenesis of Influenza	0.652	0.0351	-1	ATP6V0D2, NFKBIA, NFKBIE, PTGS2
Endothelin-1 Signaling	0.639	0.0309	1.633	CASP4, EDN1, GNAL, HMOX1, PTGS2, SHC3
3-Phosphoinositide Degradation	0.639	0.0309	-0.816	DUSP13B, DUSP8, INPP5J, MTMR7, PALD1, SGPP2
T Cell Receptor Signaling	0.627	0.026	0.535	CD28, GRAP2, H2-M2, H2-T24, HLA-A, ICAM1, ICOS, IKBKE, ITGA3, LCP2, MR1, NFKBIA, NFKBIE, RELB, SHC3, TNF
Epithelial Adherens Junction Signaling	0.612	0.0316	0.447	EGF, HGF, MET, NANOS1, SFN
Oxytocin Signaling Pathway	0.602	0.0284	1.414	CACNG8, CCL5, GNAL, IL6, MYL2, PTGS2, RELB, SHC3
ID1 Signaling Pathway	0.595	0.0299	1.633	EGF, FGR, IL6, MMP2, TGM2, TNF
Human Embryonic Stem Cell Pluripotency	0.595	0.0299	1.633	KLK3, NTRK1, PDGFB, PDGFRB, TFCP2L1, WNT10B
Sertoli Cell-Sertoli Cell Junction Signaling	0.589	0.0288	1.134	ICAM1, IL1A, IL1R1, IL1RL2, ITGA3, LAMC2, TNF
Actin Cytoskeleton Signaling	0.584	0.0287	0	EGF, EZR, ITGA3, ITGB3, MATK, MYL2, PDGFB
Inhibition of ARE-Mediated mRNA Degradation Pathway	0.578	0.0307	2.236	LTA, MAPKAPK2, SFN, TNF, TNFSF10
RHOA Signaling	0.571	0.0323	1	CDC42EP2, EZR, MYL2, RHPN2
Class I MHC Mediated Antigen Processing and Presentation	0.563	0.0267	0	ASB10, ASB4, DTX3L, FBXO32, HERC6, HLA-A, RNF213, SOCS1, TAP1, ZBTB16
CXCR4 Signaling	0.546	0.0298	-1	CXCR4, GNAL, MYL2, RHOJ, RHOV
TR/RXR Activation	0.542	0.0312	1	HP, ITGB3, MBP, ME1
Complement cascade	0.494	0.0296	-1	C5AR2, C8G, CD46, CFB
Protein Sorting Signaling Pathway	0.487	0.0281	0.447	FGR, HMOX1, PLPP3, SERPINH1, SNX29
Hereditary Breast Cancer Signaling	0.475	0.029	0	EGF, HGF, PDGFB, SFN
D-myo-inositol (1, 4, 5, 6)-Tetrakisphosphate Biosynthesis	0.46	0.0273	-0.447	DUSP13B, DUSP8, MTMR7, PALD1, SGPP2
D-myo-inositol (3, 4, 5, 6)-tetrakisphosphate Biosynthesis	0.46	0.0273	-0.447	DUSP13B, DUSP8, MTMR7, PALD1, SGPP2
Xenobiotic Metabolism General Signaling Pathway	0.445	0.028	1	GSTO2, HMOX1, NQO1, UGT1A9 (includes others)
C-type Lectin Receptors (CLRs)	0.433	0.0276	2	CLEC4E, CLEC6A, NFKBIA, RELB
Eicosanoid Signaling	0.418	0.025	1.633	ATP2A3, FPR2, GNAL, MAPKAPK2, MMP2, PTGER1, PTGS2

Glutaminergic Receptor Signaling Pathway (Enhanced)	0.416	0.0246	0.707	CACNG8, DGKI, GNAL, HMOX1, IKBKE, SCN11A, SLC38A1, SLC7A11
Superpathway of Inositol Phosphate Compounds	0.412	0.0253	-0.816	DUSP13B, DUSP8, INPP5J, MTMR7, PALD1, SGPP2
Semaphorin Neuronal Repulsive Signaling Pathway	0.406	0.0267	-1	CSPG5, ITGA3, ITGB3, MYL2
Type I Diabetes Mellitus Signaling	0.405	0.0236	1.667	CD28, FAS, HLA-A, IKBKE, IL1R1, LTA, NFKBIA, NFKBIE, RELB, SOCS1, STAT1, TNF
Huntington's Disease Signaling	0.402	0.0246	0	CAPN3, CASP4, EGF, HSPA1A/HSPA1B, NTRK1, SHC3, TGM2
Natural Killer Cell Signaling	0.388	0.0253	2	HLA-A, HSPA1A/HSPA1B, LCP2, RELB, TNFSF10
D-myo-inositol-5-phosphate Metabolism	0.384	0.0251	-0.447	DUSP13B, DUSP8, MTMR7, PALD1, SGPP2
Neddylation	0.375	0.0244	-1.633	ASB10, ASB4, DTL, FBXO32, SQSTM1, ZBTB16
Histone Modification Signaling Pathway	0.363	0.0237	1.134	DNMT3L, H2BC17, KMT5C, MMP2, SFN, TGM2, TNF
TEC Kinase Signaling	0.345	0.0225	2.121	FAS, FGR, GNAL, ITGA3, ITGB3, RELB, RHOJ, RHOV, STAT1, STAT2, TNF, TNFSF10, TXK
3-Phosphoinositide Biosynthesis	0.342	0.0239	-0.447	DUSP13B, DUSP8, MTMR7, PALD1, SGPP2
Signaling by the B Cell Receptor (BCR)	0.313	0.0235	0	CD79B, NFKBIA, NFKBIE, RASGRP3
Insulin Secretion Signaling Pathway	0.278	0.0218	1.342	CACNG8, CHRM3, FGR, KLF11, STAT1, STAT2
Chronic Myeloid Leukemia Signaling	0.269	0.0216	0.816	FAS, GLIS1, IKBKE, RELB, TNFSF10, WNT10B
MicroRNA Biogenesis Signaling Pathway	0.251	0.0214	0	EGF, HGF, HMOX1, PDGFB
Neurovascular Coupling Signaling Pathway	0	0.0172	1	ADORA2A, CACNG8, CHRM3, PTGS2
Chaperone Mediated Autophagy Signaling Pathway	0	0.0126	-2.121	ATP6V0D2, HSPA1A/HSPA1B, MMP10, MMP12, MMP13, MMP2, NFKBIA, NLRP3
NFKBIE Signaling Pathway	0	0.0178	1.414	CD40, IKBKE, LTA, NFKBIA, NFKBIE, RELB, TNF, TNFSF10
Orexin Signaling Pathway	0	0.0168	-1	ATP6V0D2, CACNG8, IL6, TNF
Generic Transcription Pathway	0	0.00935	-2	NR1D1, VDR, ZNF667, ZNF791
FC Epsilon Receptor (FCERI) Signaling	0	0.0194	1	GRAP2, LCP2, NFKBIA, TXK
Cell Cycle Checkpoints	0	0.0147	-1	ATRIP, CDC6, H2BC17, SFN
RHO GTPase cycle	0	0.0156	0.378	ARHGEF40, CDC42EP2, GFOD1, KALRN, RHOJ, RHOV, RHPN2
Mitochondrial Dysfunction	0	0.0174	-2.449	Aph1c, ARG2, CACNG8, CAPN3, Gstt1, SOD2
Role of NFAT in Regulation of the Immune Response	0	0.00967	-0.816	CD28, CD79B, GNAL, HLA-A, Ighv1-58, IKBKE, LCP2, NFKBIA, NFKBIE, RELB

CTLA4 Signaling in Cytotoxic T Lymphocytes	0	0.0164	0.632	CD28, FGR, GRAP2, H2-M2, H2-T24, HLA-A, HMOX1, ITGA3, LCP2, MR1
CD28 Signaling in T Helper Cells	0	0.0155	0	CD28, GRAP2, HLA-A, IKBKE, LCP2, NFKBIA, NFKBIE, RELB
ICOS-ICOSL Signaling in T Helper Cells	0	0.0197	-0.378	CD28, CD40, GRAP2, HLA-A, ICOS, IKBKE, LCP2, NFKBIA, NFKBIE, RELB
G Protein Signaling Mediated by Tubby	0	0.0085 5	-1	GNAL, GRAP2, LCP2, MERTK
Communication between Innate and Adaptive Immune Cells	0	0.0182	0.728	CCL3L3, CCL4, CCL5, CD28, CD40, CD79B, CD83, CXCL10, HLA-A, Ighv1-58, IL1A, IL1RN, IL36G, IL6, MR1, TLR8, TNF
Phospholipase C Signaling	0	0.0116	-0.333	CD79B, GRAP2, HMOX1, Ighv1-58, ITGA3, ITGB3, LCP2, MYL2, RALGDS, RELB, RHOJ, RHOV, TGM2
Regulation of IL-2 Expression in Activated and Anergic T Lymphocytes	0	0.0108	1.342	CD28, IKBKE, NFKBIA, NFKBIE, RELB
PKCθ Signaling in T Lymphocytes	0	0.0161	0.378	CACNG8, CD28, GRAP2, HLA-A, IKBKE, LCP2, NFKBIA, NFKBIE, RELB
PI3K Signaling in B Lymphocytes	0	0.0119	2	CD40, CD79B, Ighv1-58, IKBKE, NFKBIA, NFKBIE, RELB
Protein Ubiquitination Pathway	0	0.0183	0	HLA-A, HSPA1A/HSPA1B, MR1, TAP1, USP18
IL-4 Signaling	0	0.0086 7	0.447	COL4A2, FAS, MMP12, SOCS1, TGM2
B Cell Receptor Signaling	0	0.0126	1.633	BCL2A1, CD79B, Ighv1-58, IKBKE, INPP5J, NFKBIA, NFKBIE, RELB
Opioid Signaling Pathway	0	0.0179	0.447	CACNG8, FGR, GNAL, NFKBIA, RGS18
T Cell Exhaustion Signaling Pathway	0	0.0123	1.633	CD28, HLA-A, IL6, IRF9, LAG3, STAT1, STAT2
Synaptogenesis Signaling Pathway	0	0.019	1.633	FGR, KALRN, SHC3, SYT16, SYT7, THBS1
Systemic Lupus Erythematosus in T Cell Signaling Pathway	0	0.0187	0.302	CASP4, CD28, EZR, FAS, H2-M2, H2-T24, HLA-A, ICOS, IL6, MR1, RHOJ, RHOV
Xenobiotic Metabolism PXR Signaling Pathway	0	0.0205	1	ESD, GRIP1, GSTO2, UGT1A9 (includes others)
