# Supplementary material for: Atractylodes lancea (Thunb.) DC. [Asteraceae] Rhizome-Derived Exosome-like Nanoparticles Suppress Lipopolysaccharide-Induced Inflammation by Reducing Toll-like Receptor 4 Expression in BV-2 Murine Microglial Cells
Source: Pharmaceuticals (Basel). 2025 Jul 24;18(8):1099. doi: 10.3390/ph18081099 (PMC12389435; doi:10.3390/ph18081099)
Supplement: Supplementary file 1 [file pharmaceuticals-18-01099-s001.zip › Table S3.pdf]

Table S3a. Regulatory effect analysis of differentially expressed genes between the LPS+ELNs and LPS treatment groups

The analysis included genes with |fold change (FC)| ≥ 3 and exactTest raw p-value < 0.05.

Symbol	Expr Fold Change	Expected	Location	Type(s)
CCRL2	-11.599	Up	Plasma membrane	G-protein coupled receptor
CX3CR1	-6.341	Up	Plasma membrane	G-protein coupled receptor
MYL2	-4.442	Up	Cytoplasm	Other
MAP2K6	-4.281	Up	Cytoplasm	Kinase
TLR8	-3.981	Up	Plasma membrane	Transmembrane receptor
VIPR1	-3.67	Up	Plasma membrane	G-protein coupled receptor
P2RY12	-3.51	Up	Plasma membrane	G-protein coupled receptor
CCR3	-3.502	Up	Plasma membrane	G-protein coupled receptor
PTGER2	-3.422	Up	Plasma membrane	G-protein coupled receptor
PRKCB	-3.298	Up	Cytoplasm	Kinase
GPR183	-3.291	Up	Plasma membrane	G-protein coupled receptor
ITGB5	-3.212	Up	Plasma membrane	Other
S1PR1	-3.188	Up	Plasma membrane	G-protein coupled receptor
CCR5	-3.118	Up	Plasma membrane	G-protein coupled receptor
ITGB4	-3.052	Up	Plasma membrane	Transmembrane receptor
ADORA2B	-3.05	Up	Plasma membrane	G-protein coupled receptor
CNR2	3.086	Up	Plasma membrane	G-protein coupled receptor
MARCO	3.147	Up	Plasma membrane	Transmembrane receptor
ITGA2B	3.167	Up	Plasma membrane	Transmembrane receptor
CELSR3	3.242	Up	Plasma membrane	G-protein coupled receptor
TLR3	3.32	Up	Plasma membrane	Transmembrane receptor
GPR146	3.503	Up	Plasma membrane	G-protein coupled receptor
ITGA3	3.743	Up	Plasma membrane	Other
SUCNR1	3.762	Up	Plasma membrane	G-protein coupled receptor
SSTR5	4.596	Up	Plasma membrane	G-protein coupled receptor
PRKCG	5.771	Up	Cytoplasm	Kinase
HMOX1	6.049	Up	Cytoplasm	Enzyme

VIPR2	8.226	Up	Plasma membrane	G-protein coupled receptor
PTGER1	9.338	Up	Plasma membrane	G-protein coupled receptor

Table S3b. Regulatory effect analysis of differentially expressed genes between the ELN and control groups

The analysis included genes with |fold change (FC)| ≥ 3 and exactTest raw p-value < 0.05.

Symbol	Expr Fold Change	Expected	Location	Type(s)
TLR8	-7.281	Up	Plasma membrane	Transmembrane receptor
CCR5	-5.578	Up	Plasma membrane	G-protein coupled receptor
VIPR1	-5.259	Up	Plasma membrane	G-protein coupled receptor
CX3CR1	-4.352	Up	Plasma membrane	G-protein coupled receptor
ADRA2A	-3.949	Up	Plasma membrane	G-protein coupled receptor
ITGB3	-3.692	Up	Plasma membrane	Transmembrane receptor
GPR183	-3.333	Up	Plasma membrane	G-protein coupled receptor
CNR2	-3.308	Up	Plasma membrane	G-protein coupled receptor
GPR162	-3.194	Up	Plasma membrane	G-protein coupled receptor
C5AR2	-3.089	Up	Plasma membrane	G-protein coupled receptor
ADGRG1	-3.063	Up	Plasma membrane	G-protein coupled receptor
MYL2	-3.025	Up	Cytoplasm	Other
GPR160	-3.004	Up	Plasma membrane	G-protein coupled receptor
ITGA3	3.269	Up	Plasma membrane	Other
CHRM3	3.339	Up	Plasma membrane	G-protein coupled receptor
CCR4	3.541	Up	Plasma membrane	G-protein coupled receptor
FGR	3.879	Up	Nucleus	Kinase
VIPR2	4.181	Up	Plasma membrane	G-protein coupled receptor
GPR141	5.399	Up	Plasma membrane	G-protein coupled receptor
ADORA2A	7.697	Up	Plasma membrane	G-protein coupled receptor
SSTR5	8.311	Up	Plasma membrane	G-protein coupled receptor
HMOX1	8.927	Up	Cytoplasm	Enzyme
CALCRL	9.024	Up	Plasma membrane	G-protein coupled receptor
FPR1	11.433	Up	Plasma membrane	G-protein coupled receptor

GPR84	13.698	Up	Plasma membrane	G-protein coupled receptor
CLEC4E	13.868	Up	Plasma membrane	Other
PTGER1	19.891	Up	Plasma membrane	G-protein coupled receptor
FPR2	23.778	Up	Plasma membrane	G-protein coupled receptor
