# Supplementary figures and images for: Atractylodes lancea (Thunb.) DC. [Asteraceae] Rhizome-Derived Exosome-like Nanoparticles Suppress Lipopolysaccharide-Induced Inflammation by Reducing Toll-like Receptor 4 Expression in BV-2 Murine Microglial Cells
Source: Pharmaceuticals (Basel). 2025 Jul 24;18(8):1099. doi: 10.3390/ph18081099 (PMC12389435; doi:10.3390/ph18081099)

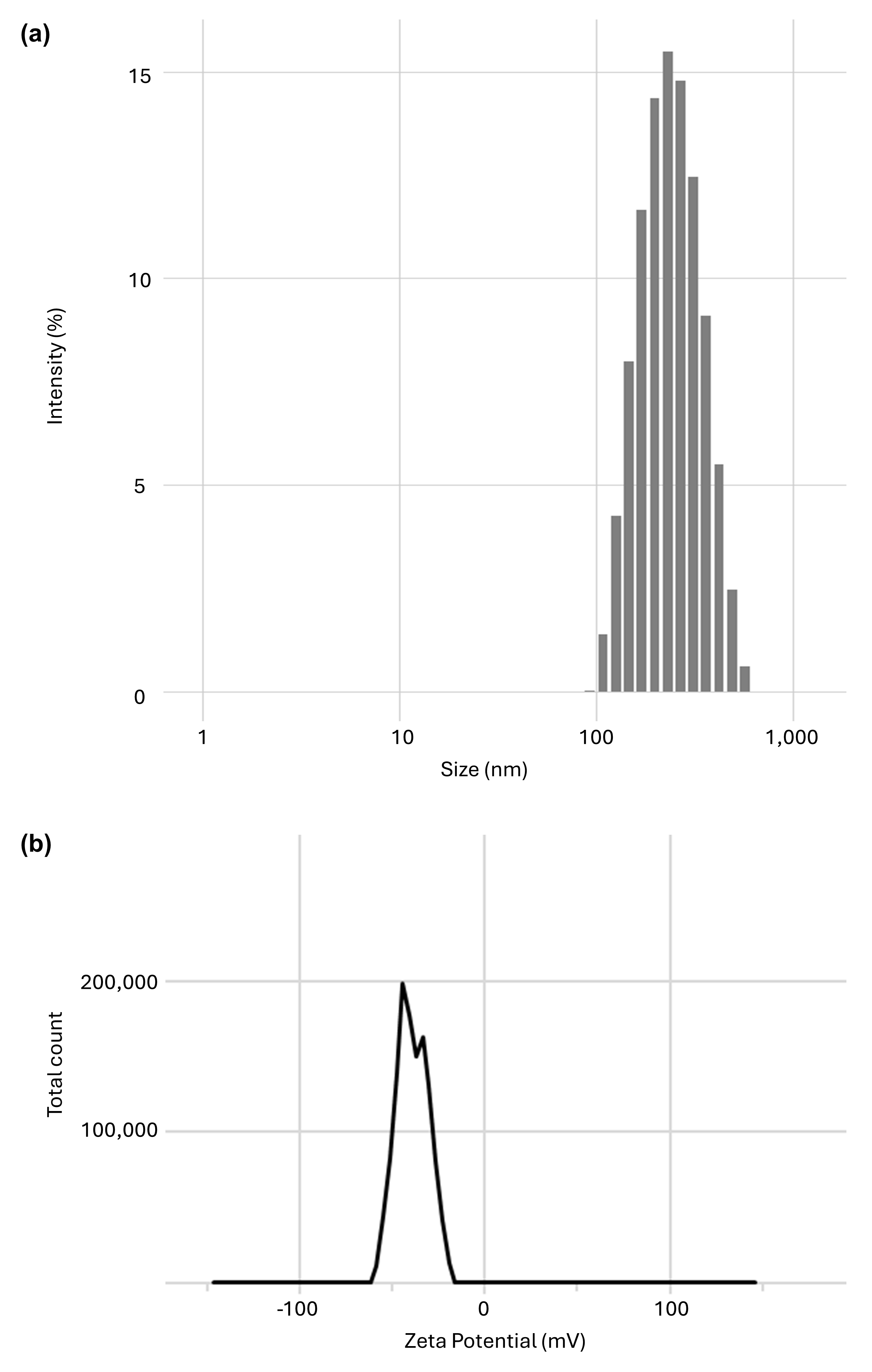

Supplement: Supplementary file 1 [file pharmaceuticals-18-01099-s001.zip › Figure S1.tif]

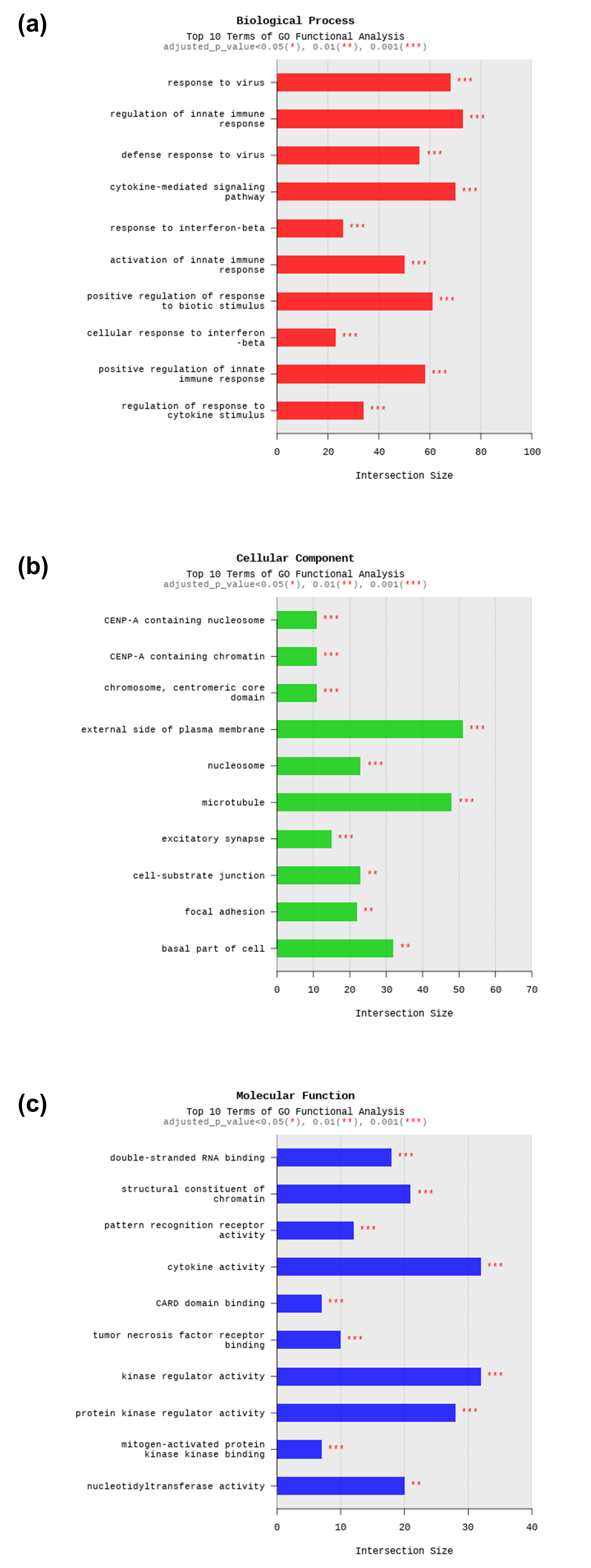

Supplement: Supplementary file 1 [file pharmaceuticals-18-01099-s001.zip › Figure S2.tif]
